# The Maternal Brain: An Organ with Peripartal Plasticity

**DOI:** 10.1155/2014/574159

**Published:** 2014-05-04

**Authors:** Katharina Maria Hillerer, Volker Rudolf Jacobs, Thorsten Fischer, Ludwig Aigner

**Affiliations:** ^1^Department of Obstetrics and Gynecology, Salzburger Landeskrankenhaus (SALK), Paracelsus Medical University, Müllner Hauptstrasse 48/Strubergasse 21, 5020 Salzburg, Austria; ^2^Institute of Molecular Regenerative Medicine, Spinal Cord Injury and Tissue Regeneration Center Salzburg, Paracelsus Medical University, Strubergasse 21, 5020 Salzburg, Austria

## Abstract

The time of pregnancy, birth, and lactation, is characterized by numerous specific alterations in several systems of the maternal body. Peripartum-associated changes in physiology and behavior, as well as their underlying molecular mechanisms, have been the focus of research since decades, but are still far from being entirely understood. Also, there is growing evidence that pregnancy and lactation are associated with a variety of alterations in neural plasticity, including adult neurogenesis, functional and structural synaptic plasticity, and dendritic remodeling in different brain regions. All of the mentioned changes are not only believed to be a prerequisite for the proper fetal and neonatal development, but moreover to be crucial for the physiological and mental health of the mother. The underlying mechanisms apparently need to be under tight control, since in cases of dysregulation, a certain percentage of women develop disorders like preeclampsia or postpartum mood and anxiety disorders during the course of pregnancy and lactation. This review describes common peripartum adaptations in physiology and behavior. Moreover, it concentrates on different forms of peripartum-associated plasticity including changes in neurogenesis and their possible underlying molecular mechanisms. Finally, consequences of malfunction in those systems are discussed.

## 1. Introduction

In all mammalian species the peripartum period is one of the most plastic periods throughout a female's life. During pregnancy and lactation, numerous changes on the physiological, cellular, and molecular level occur, which particularly distinguish a lactating mother from a nulliparous female and which prepare the female for the challenges of motherhood. Those dramatic changes in maternal physiology, plasticity of the maternal brain, and maternal behavior will not only help to ensure the survival of the offspring, but also act in concert for physiological and mental health of the mother [1–4]. However, the peripartum period represents also a time of high risk for women to develop physiological and mental disorders that are particularly associated with those peripartum adaptations. Thus, 0.5–5% of pregnant women will develop preeclampsia after 20 weeks of pregnancy [5, 6] and about 18% will be diagnosed with gestational diabetes between week 24 and 28 of pregnancy [7]. A varying high percentage of women will also be affected by perinatal mental disorders such as postpartum blues (30–75%) [8], the more long-lasting postpartum depression (10–22%) [9, 10] and postpartum anxiety (5–12%) [11, 12], or in even more serious cases postpartum psychosis (1-2%) [13]. Although some of the physiological adaptations that occur throughout pregnancy and lactation are well known, the mechanisms underlying the above-mentioned medical conditions are largely unexplored. Also, comorbidities of mental and of somatic disorders which might have originated during pregnancy are, in our opinion, underestimated. Therefore, the following review will describe the common adaptations that occur on the physiological, molecular, and behavioral level during the sensitive period around and after pregnancy and describe regulatory mechanism and potential causes for peripartum-associated disorders.

## 2. Physiological and Molecular Adaptations during the Peripartum Period

One of the first essential steps to ensure the proper development and survival of the future offspring is placentation and thereby the formation of the fetoplacental unit. Placentation is a two-stage process of coordinated invasive vasculogenesis (i.e., the formation of a branching network of vessels with chorionic villi of fetal origin) and later angiogenesis (i.e., the modification of the existing vascular network) [14–16]. The key event for a proper placental development and for pregnancy to proceed normally is the invasion of the decidual stroma of the maternal spiral arteries by the fetal cytotrophoblast [17]. After invasion, the cytotrophoblast will secrete angiogenic factors like vascular endothelial growth factor (VEGF) and placental growth factor (PlGF) [18]. VEGF is generally expressed by all cells of the fenestrated endothelium and stimulates the proliferation and survival of endothelial progenitor cells (EPCs). Given the enhanced needs in maternal blood supply during pregnancy, it is obvious that VEGF represents a crucial factor particularly during that time by stimulating vasculogenesis and angiogenesis. Inducing a generalized vasodilatation VEGF ensures the integrity of the maternal endothelium but moreover plays an important role in the proper placental development. In more detail VEGF promotes vasculogenesis and transformation of the maternal spiral arterioles from small caliber resistance vessels to large caliber capacitance vessels. This remodeling is essential for the adequate perfusion of the fetoplacental unit and consequently the exchange of nutrients, oxygen, and waste between the mother and the developing fetus (for review see [19, 20]). Although the function of PlGF during pregnancy is not fully understood yet, it seems to act to amplify VEGF-induced processes described above [21].

Aside from placental factors regulating vasculogenesis, some cytokines have been shown to be essential for pregnancy-associated events like trophoblast invasion and vasculogenesis (for review see [22]). Indeed, pregnancy is characterized by fundamental changes in maternal cytokine levels, to enable the survival of the fetus, which may be viewed as a semiallogenic graft [23]. Thus, pregnancy might be considered as a state of mild, controlled inflammation, however, ensuring the maintenance of a delicate balance between anti-inflammatory (i.e., Il-4 and IL-10) and proinflammatory (i.e., TNF*α*, IL-1, IL-6 and IL-8) cytokines.

Aside from these changes that occur during early stages of pregnancy, there are numerous adaptations at the level of the mother's brain towards the end of pregnancy and into the period of lactation. Respectively, the response of the hypothalamic-pituitary-adrenal (HPA) axis to a variety of stressors has been shown to be severely attenuated in mothers [24, 25]. The reduced peak HPA axis activity is predominantly the result of numerous central changes in excitatory and inhibitory pathways mainly within the hypothalamus. Several animal studies revealed that the pattern of excitatory inputs to the hypothalamus is altered during pregnancy [26]. Hence, a reduction in the noradrenergic tone within the nucleus paraventricularis (PVN) and a reversed opioidergic system contribute to inhibit the HPA axis activity around birth [26–29] (and see [30–34] for review). Furthermore, CRH mRNA expression in the PVN as well as CRH binding in the adenohypophysis is markedly reduced during pregnancy and lactation, leading to a diminished CRH production and release by PVN neurons [28, 35–38]. In spite of the dampened action of those excitatory systems, inhibitory systems like the oxytocin (OXT) and prolactin (PRL) system are highly activated during the peripartum period. Accordingly, OXT and PRL mRNA expression, OXT-receptor (OXT-R) and PRL-receptor (PRL-R) expression in the PVN and nucleus supraopticus (SON) [39–42], and OXT release [43–45] are increased during that time. In addition, the hypothalamic oxytocinergic system undergoes fundamental structural and functional reorganization with respect to dendritic branching and synaptic plasticity (for details, see Section 3). Clearly, peripartum-associated changes in the OXT and PRL system are essential in mediating reproductive functions such as the promotion of labour, lactogenesis, milk ejection, and maternal behavior [46–49]. Furthermore, those changes are a crucial feature to protect the late pregnant and lactating mother from overresponding to stressors (see [1–3, 34, 50, 51] for review).

Contrary to the discussed attenuated HPA axis response to physiological and psychological stressors, several mammals, including humans, rats, mice, and sheep, show an increase in basal circulating glucocorticoid levels during lactation [35, 52–54]. This lactation-associated hypercortisolism/hypercorticism might be due, at least in part, to an increased expression of vasopressin (AVP) in the PVN of the hypothalamus [37] and a simultaneous enhanced sensitivity of the pituitary to this neuropeptide [28].

## 3. Structural, Functional, and Molecular Plasticity of the Brain during the Peripartum Period

During the peripartum period the maternal brain undergoes multiple macroscopic, microscopic, cellular, and molecular changes. It is not surprising that brain regions that are particularly affected by peripartum-associated modifications are mostly those that can be summarized as the “maternal circuitry.” Some of these brain regions are crucial for the onset, maintenance, and regulation of maternal behavior (i.e., nest building, grooming, and protection of the young); others control memory, learning, and responses to fear and stress. One part of this “maternal circuitry” is the maternal motivational system, which has been nicely described by Numan in “motivational systems and the neural circuitry of maternal behavior in the rat” [55]. Briefly, Numan describes the hormonal-primed medial preoptic area (MPOA) with the adjacent bed nucleus of the stria terminalis (BNST) as central region to induce the onset of maternal behavior by suppressing fear responses to pup odors on the one hand and activating the nonspecific motivational system (i.e., the mesolimbic dopamine system) on the other hand. Brain regions involved in the avoidance of pups as seen in virgin rats are the main and the accessory part of the olfactory bulb (OB), the medial amygdaloid nucleus (MeA), the anterior hypothalamic nucleus (AHN), and the periaqueductal grey (PAG). The nonspecific motivational system is composed by the ventral tegmental area (VTA), the nucleus accumbens (NAc), and the ventral pallidum (for review see [55]). Further important brain regions that can be assigned to the “maternal circuitry” include the hypothalamic nuclei, the PVN, and SON, which are central for the regulation of anxiety and stress and the maintenance of maternal behavior (see [1, 34, 56] for review). Neurons of the hypothalamus project centrally to the limbic system, that is, the hippocampus, which interconnects to regions of the frontal lobe, that is, the medial prefrontal cortex (mPFC). Whereas the hippocampus is crucially involved in learning and memory processes not only in the context of pregnancy and lactation, the mPFC seems to be central for the perception, appraisal, and the regulation of peripartum-relevant stimuli and acts in concert with the hippocampus to regulate cognition during the peripartum period [57] ([58, 59] for review).

The following chapter concentrates on peripartum-associated changes in neural-glial interactions, synaptic plasticity, dendritic morphology, and adult neurogenesis in the selected above-mentioned brain regions.

### 3.1. Peripartum-Associated Volume Changes of the Brain

Clearly, one of the most easily observable changes that occur during the peripartum period is a change in maternal brain size, which has been shown both in humans [60] and rodents [61]. In their clinical study, Oatridge et al. recruited a total of nine healthy (control) mothers and five preeclamptic (PE) women. By analyzing brain volume* via* T1-weighted MR before pregnancy (controls), during pregnancy (controls), shortly before delivery (PE), six (PE) and 52 weeks postpartum (control and PE), they were able to show that brain size was significantly reduced whereas the lateral ventricular size was increased in both groups, respectively. This effect, which started with placental implantation and reached its maximum at term, has been shown to be even more pronounced in mothers that suffered from preeclampsia during pregnancy [60]. The observed peripartum changes in brain size seem to be interspecific, as we recently showed that absolute and relative brain weight are reduced on lactation day (LD) 14 in rats, reflecting the results in humans. In more detail, we revealed that hippocampal volume is significantly smaller in lactating compared to nulliparous females. Interestingly, the mentioned lactation-associated effect on brain weight and hippocampal volume was reversed, when rats were exposed to chronic restraint stress between LD2 and LD13 [61]. Unfortunately, the physiological importance of the above-mentioned findings in humans and rodents are not well understood at present and the underlying mechanisms still need to be elucidated.

Volume changes have not only been reported in the hippocampus, but also in other brain regions with a significant role during the peripartum period. Correspondingly, the volume of the pituitary underlies pregnancy- and lactation-associated changes. Although the pituitary enlarges during the course of pregnancy, probably due to hyperplasia of PRL cells [62], it decreases up to eight month after delivery in humans [63–65] or, respectively, seven days after delivery in rodents [66–68], when the number of PRL cells reaches prepregnancy levels [69]. Similar effects have also been observed in the MPOA and SON of pregnant/lactating rats. Respectively, cell body size (referring to soma and perikaryon) of MPOA neurons has been shown to be increased in late pregnant rats when compared to ovariectomized or diestrus rats. Interestingly, treatment with the pregnancy-mimicking regimen of progesterone and estradiol induced the same changes as seen during natural pregnancy. Given this and the fact that the area of the soma returned to prepregnancy levels with the onset of lactation [70] shows the impact of pregnancy and its attendant hormonal exposure on these changes, while during lactation the cues from pups seem to primarily maintain maternal motivation. Likewise to the pregnancy-associated changes in MPOA, the SON of lactating rats increases in volume due to hypertrophy of OXT somata and dendrites [71–73].

Morphological adaptations have furthermore been observed in another hypothalamic structure, namely, the PVN. The group around Cortés-Sol analyzed the inner capillary diameter (ICD) of 800 capillaries from magnocellular and parvocellular regions of the PVN in diestrus nulliparous female rats or at 2 weeks of lactation. In this study they were able to show that nulliparous rats presented mostly capillaries with small ICD, whereas lactating rats exhibited capillaries with larger ICD. Interestingly, the space occupied by the neurovascular compartment, such as neurons, astrocytes, and other glial cells, did not change with lactation [74], suggesting that peripartum-associated changes in angiogenesis do not exist at the level of the PVN, at least not in lactation. However, alterations with long-term impact on the neurovascular compartment might occur earlier during the peripartum period. Respectively, human studies have shown a significant increase in the number of circulating EPCs with the progression of pregnancy [75], an effect that was absent in women with preeclampsia [76, 77]. This is an important finding, as the peripartum-associated changes in EPCs and the microvasculature of the PVN might play an important role in enhanced availability of the neurohypophyseal hormones AVP and OXT during the peripartum period by increasing their cytoplasmatic transport from the luminal to the abdominal side of the membrane. Indeed, OXT plasma concentrations have been shown to be diminished in women with gestational hypertension [78]. As hormones like AVP and OXT have been shown to be implicated in mechanisms of cell volume regulation in rodents [79], it might be that PVN OXT neurons modulate their own capillary blood supply and* vice versa*. Given the fact that the OXT system in the hypothalamus undergoes intriguing morphological plasticity during the peripartum period, which will be discussed in one of the following paragraphs, such a mechanism seems to be indeed very likely.

### 3.2. Peripartum-Associated Receptor Plasticity in Different Relevant Brain Regions

During the peripartum period the maternal brain undergoes marked changes in receptor expression. Unsurprisingly, brain regions and neuronal systems that are affected the most by these alterations are those known for their importance in different aspects of maternal behavior. With their rat model of high licking/grooming (LG) and low LG rats, Meaney and coworkers have significantly contributed to a better understanding of the correlation between maternal care and receptor plasticity in different neuronal systems. In a couple of elegant studies they did not only reveal that an increase in estrogen-receptor *α* (ER*α*) expression in the MPOA on LD6 was correlated with an increased level of LG behavior in the high LG group, but also that the maternal behavioral phenotype was epigenetically transmitted over generations by a cytosine methylation process across the ER*α*1b promoter [80, 81]. Interestingly, the level of ER*α* expression seems to occur as a function of reproductive experience as seen in mice, rats, and sheep [82–84]. In more detail, ER*α* expression in the MPOA, but also the MeA, the PVN, and SON has been shown to be increased four days before parturition in multiparous ewes [84], which might explain the increased estrogen responsiveness to induce maternal behavior in multiparous dams [85]. The effect of parity on ER*α* expression seems to be long-lasting, as an increase in the MPOA and MeA has been observed in middle-aged cycling females several month after pregnancy [82].

Similar to ER*α*, OXT-R expression levels have been found to be increased in several distinct “maternal” brain regions like the MPOA, VMH, BNST, lateral septum (LS), central amygdala (CeA), and the PVN of rodents (see [86] for review). The observed alterations in OXT-R distribution pattern reflect the crucial role of the OXT system to orchestrate different aspects of maternal behavior, including maternal care. Indeed, there is a close relationship between maternal care and OXT-R mRNA expression levels in those brain regions. Accordingly, dams characterized by a high maternal responsiveness to pups (i.e., high LG) have significant higher OXT-R levels in the MPOA, the CeA, the LS, and the BNST compared to mothers that show low levels of LG behavior [87]. Importantly, lowering OXT-R expression by repeated exposure to restraint stress between pregnancy day (PD)15 and PD21 or inhibiting OXT-R action by administration of an OXT-R antagonist on LD3 eliminated the described differences in maternal behavior [87, 88]. Similarly, hypothalamic OXT-R expression has been shown to be essential for the lactation-associated anxiolysis, as central application of an OXT-R antagonist in the PVN increased anxiety in lactating but not virgin or male rats [44]. Given the fact that both OXT and PRL are key players during the peripartum period, acting synergistically to regulate maternal care, anxiety, and stress, it is not surprising PRL-R expression is comparably altered during that time. An upregulation of PRL-R mRNA has been shown in many brain regions known for their importance in regulating maternal behavior like the MPOA, the BNST, and VMH during different timepoints within the peripartum period (i.e., PD12, 2 h postpartum, LD7-10) [89, 90]. Marked changes have been found in the PVN and the SON of rats, where the proportion of OXT neurons expressing the long form of the PRL-R was significantly increased during pregnancy and lactation [41], providing evidence that PRL directly and specifically regulates the activity of OXT neurons. Likewise to the above described studies regarding the OXT system, lowering PRL-R activity by chronic administration of PRL-R antisense oligonucleotide increased anxiety-related behavior and inhibited the PRL-mediated attenuated responsiveness of the HPA axis, thus explaining the parallel stress-hyporesponsiveness during lactation [91, 92]. Aside from the regulatory role of receptor plasticity in the stress-induced HPA axis activity, peripartum-associated alterations in receptor expression also seem to be essential in the dampened sensitivity of the adrenocortical negative feedback during that time. Indeed,* in vitro *binding assays using 3[H] dexamethasone as radioligand revealed a reduced glucocorticoid receptor binding capacity in the hippocampus of rats during the first two weeks of lactation [93].

Aside from the onset and display of maternal care, altered anxiety, and stress responses during lactation, the expression of maternal aggression is an important feature ensuring sufficient protection and thus survival of the offspring. Studies in rats revealed that peripartum-associated fluctuations of the vasopressin receptor V1a (V1a-R) in brain regions of the “maternal circuitry” are substantial for the timely and fine-tuned expression of maternal aggression. Accordingly, the level of V1a-R binding in the BNST, MPOA, LS, and CeA has been shown to be positively correlated with the level of aggressive behavior throughout pregnancy, parturition and lactation [94]. Further proving the specific importance of the peripartum-associated V1a-R upregulation in the control of maternal aggression, bilateral infusion of a selective V1a-R antagonist into the BNST (daily between LD1 and LD6) significantly reduced maternal aggression, while it did not affect the frequency of arched back nursing [95].

In summary, the multiplicity of studies revealing changes in receptor expression of hormonal systems in specific maternal brain regions highlights the importance of these changes during the peripartum period. Together with other peripartum-associated forms of plasticity as will be discussed in the following sections, such changes in receptor expression act synergistically to positively influence the behavioral repertoire, physiological and psychological wellbeing of the mother, and thus survival of the offspring.

### 3.3. Peripartum-Associated Synaptic Plasticity of the OXT System in the Hypothalamus

Classically, synaptic plasticity such as long-term potentiation or depression is defined as long-lasting functional modification within preexisting synapses [96]. However, synaptic transmission can also be significantly altered by structural synaptic plasticity. During the peripartum period, this form of synaptic plasticity occurs in the hypothalamo-neurohypophysial SON and magnocellular PVN, when OXT neurons undergo an extensive neuronal and glial remodeling [97]. Thus, it has been shown that under conditions that stimulate OXT release, such as parturition and lactation, there is an increase in the numerical density of axosomatic and axodendritic synapses on OXT neurons in the SON as seen in tissue of rats that have nursed their pups for 11 days [98]. These changes, which occur rapidly within 24 h of stimulation [99] ([100] for review), are concomitant with a glial retraction of OXT somata and dendrites leading to a juxtaposition of a significant portion of the plasmalemma [73, 101, 102]. The reduction in the astrocytic coverage of postsynaptic elements is an essential prerequisite for the above mentioned formation of new synaptic contacts during the peripartum period and alters neuronal function directly* via* the modification of synaptic transmission and indirectly by preparing neuronal surfaces for synaptic turnover. Once stimulation is over, astrocytic processes will again cover the OXT surfaces as seen under basal conditions in different adult neuronal tissues (see [103] for review). Aside from the peripartum-associated changes in the number of synapses, there have also been rodent studies revealing an increase in the number of shared synapses in OXT neurons [71, 97, 100, 102], further amplifying the synaptic input. As well as for the above-mentioned changes in the number of spines, the synaptic coupling of OXT neurons takes place rapidly within two hours after stimulation [104]. Another important aspect of synaptic plasticity in the hypothalamus that occurs with the onset of lactation is a remodeling of afferent inputs that control the activity of the OXT system. Although under basal (i.e., nonlactating) conditions about 35% of synapses in the SON are GABAergic [71], 22% are glutamatergic [71], and 10% are noradrenergic [105], this distribution changes during the stimulatory influence of lactation. Hence, analysis of brain sections from lactating rats that had suckled litters for at least 10 days revealed that there is an increase in axosomatic and axodendritic GABAergic synaptic contacts, accounting for about 50% of all SON synapses in lactating rats [71, 98]. The origin of the GABAergic input to the magnocellular neurons arises from local interneurons [106, 107], as well as from adjacent hypothalamic areas [108, 109]. Although, the origin of the glutamatergic afferents is comparable with those of GABA, morphological remodeling is less pronounced in glutamatergic synapses with only a 3% increase compared to basal conditions [71, 102]. Although, noradrenergic innervation of OXT and AVP neurons is equal in nulliparous females, there is a significant increase in noradrenergic innervations of OXT neurons during lactation [105]. The fact that fine-tuned morphological plasticity in the OXT system appears fairly rapid and disappears once stimulation is over raises of course the questions of its functional implications. It has been speculated that the increased number of synaptic contacts as well as an increase in both inhibitory and excitatory inputs is an important mechanism for the unique pattern of electrical activity that characterizes OXT neurons during lactation, that is, their pulsatile firing ability, leading to the bolus release of OXT (see [110, 111] for review). Furthermore, increased inhibition by GABA might be needed to ensure that activation of OXT neurons only occurs by lactation-relevant stimuli, but not others. Indeed, this possibility seems to be likely, as stress-induced stimulation of OXT neurons by injection of hypertonic saline has been shown to be reduced during the peripartum period [112].

### 3.4. Peripartum-Associated Neuronal Plasticity in the Hippocampus and the SVZ/OB

Another important contribution to maternal neuroplasticity is provided by the mechanism of adult neurogenesis. Although the production of new neurons throughout adulthood has been suggested in several brain structures including the neocortex, piriform cortex, amygdala, striatum, substantia nigra, dorsal vagal complex, and the hypothalamus of mammals (see [113, 114] for review), peripartum-associated changes have only been revealed in the two main neurogenic regions, namely, the subventricular zone (SVZ) of the lateral ventricles and the subgranular zone (SGZ) of the hippocampal dentate gyrus (DG). The SVZ gives rise to neuroblasts migrating a fairly long distance* via *the rostral migratory stream (RMS) to the OB, where they differentiate in one of the two main types of interneurons in the OB, granule cells (GC), or periglomerular neurons. Stem cells in the granule cell layer of the DG only migrate a short distance after proliferation and differentiate to one of the three main cell types in the hippocampus, neurons, astrocytes, or oligodendrocytes.

To date, the functional implication of adult neurogenesis during the peripartum period is not fully understood; however, given the fact that newly generated cells fully integrate in mature preexisting circuits it is likely that they significantly contribute to an enhanced plasticity and responsiveness to specific stimuli in a new environment with changing demands during this susceptible time. The following paragraph will discuss the state of the art of peripartum-associated alterations in adult neurogenesis and their possible contribution to maternal behavior and* vice versa*. The underlying regulatory mechanisms will be highlighted in Section 5.

Numerous studies have been performed during the last decade, increasing the understanding of the mechanisms controlling adult neurogenesis and its functional implications during pregnancy and lactation. Taking the broad consensus of all these studies it appears that the peripartum-associated changes in adult neurogenesis are not only dependent on the neurogenic region looked at, but also are species- and time-dependent. Hence, cell proliferation in the SVZ has been shown to be increased on PD7 in mice, but not at later time points during pregnancy (i.e., PD14 and PD21) [115, 116], whereas in rats the complete opposite picture occurred [117]. The observed differences might be explained by methodological differences, that is, the number of BrdU injections and the amount of BrdU injected [118]. However, the species-dependent discrepancy might also be due to the level of functional importance the olfactory system plays in relation to maternal behavior in both species. The observed increase in cell proliferation on PD7 might lead to the integration of a greater number of newly generated interneurons at parturition, when the olfactory demands are high and might thereby contribute to the onset of maternal behavior in mice. Inhibition studies seem to be an applicable, insightful tool to investigate the importance of SVZ/OB neuroplasticity in relation to maternal behavior. However, as the methods disrupting neurogenesis differ a lot with respect to their specificity and thus to their extent in outcome, care must be taken when interpreting their results. Accordingly, it is not surprising that partially controversial results that are achieved are dependent on the approach used. By genetically ablating newly born neurons in the forebrain of mice Sakamoto et al. were able to show that new mothers showed abnormal maternal behavior and significant impairments in retrieval behavior, leading to the death of pups within 24–72 h after delivery [119]. Similar results were achieved by the group of Larsen who used the antimitotic drug bromocriptine to inhibit SVZ mitogenesis in PD7 mice, consequently reducing the number of neurons in the OB on LD2. Although, retrieval behavior in the home-cage was unaffected, pup retrieval was severely impaired when the mothers were tested in a novel environment [116]. It has to be kept in mind though that both genetically targeted ablation and the use of antimitotic drugs lack specificity of targeting neurogenic regions. Thus, it is not precluded that the observed changes in behavior seen in those studies are due to disruption of processes in other neurogenic regions like the hippocampus. Contrary to the above-mentioned results, a study using focal gamma irradiation which specifically disrupts neurogenesis in the SVZ/OB circuitry did not result in an impairment of maternal behavior [120]. Given the fact that adult-born neurons are fully responsive to odor stimuli at two weeks of age [121] and the fact that olfaction plays a key role in the establishment of maternal behavior, learning and bonding in mammals (including mice, rats, sheep, goats, and humans) [122] (and see [123, 124] for review) suggests that the integration of adult-generated neurons in the preexisting olfactory circuitry might be rather required for odor learning and distinguishing than for maternal behavior itself. However, given the varying effects observed, further studies are highly desired to finally elucidate the contribution of peripartum-associated changes in SVZ/OB neurogenesis with relation to maternal behavior.

**Figure 1:**
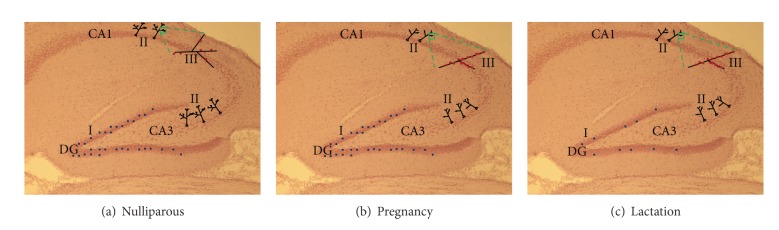
Graphical presentation of hippocampal morphology in the hippocampal subregions dentate gyrus (DG), CA1, and CA3 in (a) nulliparous, (b) pregnant, and (c) lactating females; I: cell proliferation/survival, II: dendritic length and complexity of pyramidal neurons, and III: spine density on pyramidal neurons.

Although, the importance of pregnancy-stimulated neurogenesis in the SVZ with respect to lactation has been beautifully demonstrated by the studies of Shingo et al. [115], there is no proof so far that lactation itself would affect adult neurogenesis in the olfactory system in rodents. However, it seems that pup presence/maternal behavior has the potential to induce lactation-associated changes in SVZ neurogenesis in sheep and rodents. Respectively, a stimulatory effect of interaction with the young has been observed in sheep during the early postpartum period [125]. Comparable observations were made by Furuta and Bridges in their experiments in rodents, when they exposed nulliparous female mice daily to foster pups in order to induce maternal behavior. When they compared maternal nulliparous females with those in which daily fostering was not efficient to induce maternal behavior, they found an increase in the number of BrdU+/NeuN+ double-labeled neurons in the SVZ of the former group [126]. Interestingly, comparing studies analyzing olfactory and/or hippocampal neurogenesis suggests that parturition and interaction with the young affects both cell proliferation and cell survival in a different manner across the neurogenic zones. Thus, contrary to the documented results regarding adult neurogenesis in the OB, there is little or conflicting evidence that pregnancy is associated with changes in hippocampal neurogenesis. Although BrdU+ cells and BrdU+/DCX+ cells have been shown to be reduced in mice on PD14 [127] and differentiation and migration of adult generated neurons have been shown to be increased in a rat model of hormone-simulated pregnancy [128], no changes in cell proliferation have been reported in mice and rats during early [115, 117, 129] or other late stages [117, 129] of gestation. However, there is a significant change in hippocampal neurogenesis during the postpartum period. In all species studied so far, cell proliferation is severely diminished during early and midlactation, an effect that is restored by the time of weaning [61, 125, 130–132]. Similarly, one-week survival of newly born cells has been shown to be decreased, whereas two-week survival was unchanged in lactating rats [131]. Whether cell survival is altered at parturition or the first days postpartum, as seen in the OB is not known so far. In summary it seems that maternal experience, but particularly the presence of pups, has a suppressive, albeit temporary effect on hippocampal neurogenesis, as nicely revealed in lactating female rats. Respectively, removal of pups immediately after birth, thus preventing nursing and the associated rise in CORT levels, restored cell proliferation in dams to the level of nulliparous females [131]. Interestingly, the observed effect may not be based on nursing alone, as similar results have been observed in male California mice, a species known to show biparental care. In more detail, Glasper et al. showed that hippocampal neurogenesis was reduced at the time of weaning in parenting fathers when compared to nonparenting fathers [133]. However, the effect of parenting on hippocampal neurogenesis in males seems to be highly species dependent as other studies in prairie voles and inbred C57BL mice showed a stimulatory effect of parenting on neuron production [134, 135]. Given the fact that environmental enrichment is typically believed to have beneficial effects on neuroplasticity (for review see [136]) and the fact that maternal/paternal experience implicates such enrichment, the results regarding a reduction in hippocampal neurogenesis at the first glance seem to be surprising. However, aside from pup presence the main stimulus for the observed changes are the actual physiological conditions associated with the peripartum period, as nulliparous rats that were exposed to pups actually showed an increase in cell proliferation [132]. The detailed molecular mechanism underlying peripartum-associated alteration in SVZ/OB and hippocampal neurogenesis will be the subject of Section 5 and we would kindly refer the reader to the respective section. To date the exact functional contributions of maternal experience-induced changes in hippocampal structure are largely unknown; however, the fact that being a mother can remodel neuronal systems in such extent indicates that they play an important role during this sensitive time frame and might be fundamental for the maternal physiological and mental health.

(see also Figure 1 for graphical presentation of peripartum-associated changes in hippocampal neurogenesis.)

### 3.5. Peripartum-Associated Changes in Dendritic Morphology and Spines in Different Relevant Brain Regions

The effects of parenting on neuronal plasticity are not only limited to alterations in cell proliferation and survival, as discussed above, but also extend to complex morphological changes, including alterations in the number and density of spines, as well as dendritic architecture.

Over the last decades, several studies in rodents revealed peripartum-associated alterations in different relevant brain regions like the hippocampus, the OB, the mPFC, and the MPOA, as well as the PVN and the SON of the hypothalamus. Thus, pregnancy has been associated with a reduced complexity of CA3 pyramidal neurons of the hippocampus as seen in rats [129]. Although CA1 pyramidal neurons do not show these pregnancy-dependent changes [129], they are altered during lactation as after weaning Respectively primiparous rats exhibit a reduction in dendritic length and fewer dendritic branch points on pyramidal CA1 and CA3 neurons, when compared with nulliparous or multiparous females [137]. The underlying mechanisms for the observed shrinkage are not well understood; however, it might be due to the lactation-associated hypercorticism, as studies in male rats revealed dendritic atrophy in hippocampal pyramidal cells after chronic stress-induced elevation of glucocorticoids [138]. Interestingly, not only the dendritic architecture seems to be modulated by motherhood, but typical neurogenic regions, like the hippocampus and the OB, also undergo remodeling of dendritic spines. Kopel et al. performed numerous elegant studies in mice revealing that dendritic spines of adult born granule cells (abGCs) underlie changes during lactation that might contribute to olfactory encoding during that time and thereby have a direct impact on mother-pup interactions. By injecting a lentivirus that encoded synaptophysin which was fused to GFP into the stem cell niche of the SVZ, they were not only able to show that abGCs of lactating females show an increase in the density of presynapses, but also that their spine stability and integration into the bulbar network are increased during motherhood [139]. Similar changes seem to occur in the hippocampus, where an increase in spine density on apical dendrites of CA1 pyramidal neurons and DG granule cells has been observed in late pregnant and postpartum rats [131, 140]. While the increased spine density during pregnancy seems to be mediated by estrogen as suggested by studies in nulliparous rats that showed comparable changes after a hormone-stimulated pregnancy [141], such a regulation is unlikely during lactation as estrogen levels drop after parturition. Thus, it might be that pregnancy-generated spines either maintain throughout lactation or that environmental enrichment in form of maternal experience is beneficial for dendritic spine growth as seen before in humans [142] (and see [136] for review). Given the fact that dendritic arborization of these CA1 neurons has been shown to be diminished during lactation as mentioned before, the observed increase in dendritic spines might be seen as compensatory mechanism to increase the efficacy of synaptic inputs despite the reduced dendritic length and number of branching points in those dams. Definitely, the increase in spine density is only transient during lactation and dependent on the presence of pups, as there is a decline in primiparous rats after weaning [137]. However, the persistence of dendritic spines might also depend on the reproductive experience, as multiparous females have been shown to have more dendritic spines after weaning compared to primiparous mothers [137, 143]. Given the fact that the CA1 region of the hippocampus is connected with the mPFC, the before mentioned peripartum-associated alterations in hippocampal spine density may also implicate changes in the forebrain. Indeed, dendritic architecture of the mPFC cortex undergoes an intense remodeling during lactation. By analyzing layers 2 and 3 pyramidal neurons of late lactating rats (LD20-24) Leuner and Gould were able to show that dendritic length and branching, as well as the number of spines on apical and basal dendrites, were increased, which directly coincided with an improved behavioral flexibility as assessed by an attentional set shifting task [57], a task which has been previously shown to depend on the PFC [144]. The onset of such dendritic changes in the mPFC seem to occur even earlier during lactation, as we made similar observations when we compared 3D reconstructions of neurons in the infralimbic mPFC of early lactating (LD3-5) and nulliparous rats (own unpublished results). These results are astonishing, given that the adverse hormonal environment during lactation and high glucocorticoid levels have been associated with a diminished dendritic architecture and spine density in the mPFC of nulliparous rats [145, 146]. However, the effect of altered glucocorticoid levels during lactation might be highly region-specific as an increase in mushroom spines on apical and basal dendrites has been reported in lactating rats that have been chronically treated with high doses of CORT (40 mg/kg/day) between LD2 and LD23 [147]. Either way, it seems that the postpartum period or the associated increase in neuronal activity by the stimulatory effect of pup-contact [148, 149] is able to buffer against the adverse basal hypercorticosterone environment and promotes dendritic growth in the mPFC. One hormone that might play a key role in this respect is OXT, which is known to be released during mother-infant contact in humans and rodents (for review see [150]) leading to an activation of the prelimbic prefrontal cortex [148]. We have previously shown that chronic stress during pregnancy in rats impairs the peripartum-associated increase in OXT activity in the PVN [151]. Given the fact that a reduction in the mean apical dendritic length in the mPFC has been associated with increased levels of anxiety in male rats [152] and chronic stress has been shown to increase anxiety during lactation [151], it would be interesting to see if dendritic morphology is a prerequisite of the peripartum-observed anxiolysis and if OXT might play a role in dendritic remodeling of the mPFC during that time.

As already discussed in one of the previous sections, the OXT system of the hypothalamus undergoes tremendous peripartum-associated changes with respect to synaptic plasticity. Aside from those changes in synaptic transmission, neuronal-glial remodeling, and rearrangement of synaptic inputs, morphologic changes of OXT neurons have been reported in lactating rodents. Dendritic trees of the magnocellular neurons of the SON undergo cytoarchitectonic reorganization during lactation that are very much evocative of those reported in the hippocampus as described before and can effectively contribute to lactation-induced neuronal plasticity by altering the electrical and integrative properties of OXT neurons during that time. In a number of elegant studies in rats Stern and Armstrong revealed that the dendritic trees of OXT neurons in the SON shrink during lactation. The reduction in total dendritic length was accompanied by a loss of dendritic branching on middle order branches (100–200 *μ*m from soma) of these neurons [153]. Given the broad consensus that an increased dendritic arborization and length usually leads to beneficial outcomes in behavior (i.e., reduced anxiety and increased cognitive performance) [57, 152], these results might initially be surprising; however, the observed changes might contribute to the specific properties of the OXT system during lactation. Hence, a diminished branching would increase the postsynaptic space and thereby alter the electrical properties and the efficacy of synaptic inputs, which reach the soma to determine the output of the neuron [154]. This in fact might allow the high-frequency bursting activity pattern typically seen in OXT neurons during lactation leading to the milk ejection reflex [155]. Moreover, the dendritic shrinkage might involve a loss in synaptic contacts, leading to neurons that will deal with fewer, but more specific inputs in a more specific way. Indeed, the response of OXT neurons to nonspecific/not pup-related stimuli has been shown to be reduced during lactation [156, 157].

We would also like to refer the reader to Table 1 that summarizes the findings discussed in this section.

## 4. Behavioral Adaptations during the Peripartum Period

During the peripartum period there is a whole set of cognitive and behavioral changes. Clearly, the display of maternal behavior, which also includes the expression of maternal aggression, is one key behavioral change that occurs during the peripartum period. As most mammalian species are not spontaneously maternal, the elevated availability of “maternal” neuropeptides like OXT [3, 158], PRL [91, 159], as well as AVP [95, 160] and their respective receptors [39, 42, 90, 94, 161], is a prerequisite for the onset and maintenance of the complex repertoire of maternal behaviors. Peripartum-associated alterations in those brain factor systems not only are of vital importance for maternal behavior, but also they act in concert to decrease anxiety during lactation [37, 44, 91, 116], as revealed by inhibition studies in rats [44, 91, 116, 162] (and see [1] for review). The peripartum-associated anxiolysis, which can also be observed as increased calmness in breast-feeding mothers [25], is known to be accompanied by a coherent increase in aggression during lactation [163–165]. In this context, the CRH system, whose activity is decreased during lactation as discussed before, has been shown to be of relevance. Indeed, increasing CRH availability results in lower levels of maternal aggression in rodents [166, 167]. Thus, it appears that the reduced activity of the CRH system not only is involved in the stress-hyporesponsiveness during lactation, but also is an important regulator of maternal aggression and anxiety. It is likely that the peripartum-associated changes in anxiety and aggression not only are essential to ensure protection and survival of the offspring [168, 169], but are also fundamental for maternal mental health.

Aside from the above-mentioned changes in maternal behavior, that is, maternal anxiety and maternal aggression, the peripartum period is a time of alterations in cognitive abilities, particularly those that are associated with spatial learning, memory, and navigation as revealed in several animal and human studies using different tests to assess cognitive performance. The outcome, that is, an increase or decrement in cognitive abilities, seems to strongly depend on a variety of factors like species, parity, fetal sex, time of assessment, and test used (see [170, 171] for review). Studies in rodents indicate that early and midpregnancy are generally associated with an increase in spatial working and reference memory in a couple of hippocampus-dependent tasks. Respectively, spatial memory is increased in dams as revealed by the Morris-Water-Maze [172–174] or Object-Placement-Task [175, 176]. The observed improvements in cognitive ability seem to occur between PD7 and PD10, as pregnant rats have been shown to outperform nulliparous rats in the Morris-Water-Maze within this timeframe. However, the initial procognitive effects of early pregnancy are not continuous as they are followed by a decrement in cognitive abilities in late pregnancy into early lactation, a time when the mother's brain is usually referred to as the “pregnant brain.” Accordingly, human mothers show a significant decrease in working memory, verbal memory, word recall, visual memory, spatial memory, explicit and implicit memory, and attentional processes [177–182] and even self-rate them to have a poorer memory compared to the prepregnancy state [183]. The results from clinical research are in line with those from rodent studies, revealing a reduced performance in the Morris-Water-Maze on LD1 and LD4 [130]. Interestingly, deficits in memory are dependent on fetal sex, as human mothers being pregnant with a male fetus outperformed those pregnant with a female fetus in a working memory and spatial ability task [182]. Furthermore, an evolutionary component is likely to drive the observed changes in cognition, at least in part. Thus, while visual memory is diminished in human mothers, recognition memory is unaffected [184, 185], indicating that the latter might be ethologically more important and therefore be maintained. After all, also the changes in late pregnancy and early lactation are only transient and give the way to cognitive improvements being effective throughout the late postpartum period and weaning and long into the process of aging even under conditions of stress. Respectively, late lactation has been associated with an increase in spatial working memory in a Land-Maze-Task [186], the Morris-Water-Maze [187], and Radial-Arm-Maze [188, 189], as well as memory in an Object-Placement-Task in rodents [190]. In contrast to those tasks requiring spatial memory, tests assessing nonspatial memory did not reveal changes during the postpartum period in rodents [187], suggesting that the peripartum-associated changes in cognition play a predominant role in effective and efficient foraging, which is highly dependent on the spatial ability of the mother. Similar to the findings in rodents, human studies found verbal, semantic, and working memory, as well as attention, to be improved even two years after delivery [191]. Interestingly, maternal experience seems to have a long-term protective effect on learning, even under conditions of stress as revealed by studies in rats by Maeng and Shors [192]. They showed that exposure to an acute stressor suppressed learning in a classical eyeblink conditioning in virgins, but not in females that had been mothers five to nine month before the testing [192]. The peripartum-associated improvement in cognitive function seems to require structural reorganization of different brain regions like the hippocampus or the mPFC. Indeed, postpartum rats (LD20–LD24) showed an improved performance in an attentional set shifting task that was coincident with an increase in dendritic spines on pyramidal neurons of the mPFC [57]. The mentioned improvement was selective to extradimensional set shifting, which is known to require the mPFC, but not discrimination learning, intradimensional set shifting, or reversal learning [57]. Similarly, the observed increase in hippocampal-dependent learning during motherhood [188, 189] might depend on dendritic remodeling in CA1 and CA3 regions of the hippocampus [137]. Given the well-known link between spatial learning/memory and hippocampal neurogenesis [193, 194] and the fact that hippocampal neurogenesis is altered during the peripartum period as described before, it might be hypothesized that the observed alterations in cognition might be based on this relationship. Assessing hippocampal neurogenesis, spatial reference, and working memory in nulli-, primi-, and multiparous rats, Pawluski et al. nicely revealed that parity is an essential factor affecting both parameters. Nevertheless, it seems that neurogenesis might only play a minor role in peripartum-associated changes in cognition, since cell survival is diminished in primiparous rats across the postpartum period, while such animals outperform nulliparous control animals in cognition [132, 188, 189]. Other evidence from rodent and human studies suggests that parity might be an important factor influencing cognitive ability in age, although the outcome is controversial. In rodents, multiparity has been linked to better performance in several cognitive tasks when tested between six and 24 month of age that is long past their reproductive experience. Respectively, multiparous rats outperformed nulliparous and/or primiparous rats in a spatial memory task, object recognition/placement, and spatial memory task [195, 196]. Importantly, multiparity and spatial learning/memory performance in the Morris-Water-Maze was negatively correlated with the level of immunoreactive amyloid precursor protein (APP), a marker of neurodegeneration and cognitive loss [195]. These results are in contrast to studies in humans indicating that the number of lifetime pregnancies is positively correlated with the risk to develop early onset Alzheimer's disease [197].

## 5. Molecular Processes That Underlie the Observed Peripartum Changes and Consequences of Malfunction

As illustrated throughout this review, there are numerous essential alterations in physiology, behavior, and neuroplasticity during pregnancy and lactation. Although, some of their underlying mechanisms have already been addressed in the respective section, in this chapter we would like to highlight some molecular mechanisms in more detail that significantly contribute to the peripartum-observed changes. Moreover, we will outline the consequences of malfunction in such systems for the pregnant or lactating mother.

As discussed before, there are some striking adaptations of the hypothalamic OXT system during the peripartum period. Given the extent of those changes and the importance of the OXT system during that susceptible time, the question of their underlying molecular mechanisms is raised. One molecule that has been shown to be a key player in the peripartum-observed hypothalamo-neurohypophysial structural synaptic plasticity is PSA-NCAM. This neural cell adhesion molecule is abundantly expressed on astrocytic surfaces of rodent glial cells in the SON during the peripartum period [198, 199] and is an important regulator of cell adhesion and contact-dependent cell surface interactions (for review see [200, 201]). Indeed, PSA-NCAM is an essential prerequisite for the structural synaptic changes in the magnocellular nuclei of the hypothalamus, as revealed by enzymatic perturbation experiments. Thus, if endoneuraminidase was microinjected in the hypothalamic magnocellular nuclei* in vivo *and thereby enzymatically removing PSA from NCAM, neuronal, glial, and synaptic remodeling typically associated with lactation was inhibited [198]. Interestingly, OXT itself seems to stimulate the PSA-NCAM regulation of its own synaptic plasticity during the peripartum period, as ICV administration of OXT induces changes similar to those seen under physiological stimulation, that is, lactation, when OXT levels are high [202, 203]. Hence, OXT which is dendritically released during parturition and lactation [43] may play a major role to induce synaptic plasticity in the hypothalamus during the peripartum period.

Although PSA-NCAM has some regulatory influence on SVZ/OB neurogenesis by stimulating tangential migration from the SVZ to the OB in rats and mice [204, 205], the main regulatory factors for neuroplasticity in those regions are others. Shingo et al. were the first to elucidate that the endocrine state of an animal can actually influence the rate of neurogenesis. In more detail they were able to show that the rise in central PRL levels during pregnancy is the main driving factor for the increase in SVZ proliferation on PD7 and consequently the increased number of OB neurons two to four weeks later [115]. Indeed, lowering serum PRL levels by administration of bromocriptine during pregnancy led to a decrease in cell proliferation and cell survival in the SVZ/OB [115]. Moreover, they revealed that the action of PRL is mediated via PRL-R in the SVZ, as mice expressing the heterozygous form of the PRL-R (PRL^+^/^−^) showed lower levels of cell proliferation on PD7 when compared with mice expressing the homozygous form of the receptor (PRL^+^/^+^) [115]. Interestingly, hippocampal neurogenesis was unaffected by bromocriptine treatment [116], suggesting that maternal experience can have opposite effects on the same form of structural plasticity in two different brain regions via different distinct regulatory mechanisms. Although the factors regulating hippocampal neurogenesis during the peripartum period are still barley known, several studies significantly contribute to a better understanding of peripartum-associated changes in this neurogenic process. Thus, Rolls et al. nicely shed some light on one possible mechanism underlying the reduced hippocampal cell proliferation that they observed between PD11 and PD12 in mice [127]. Given the fact that the immune system has been shown to be an important regulator of neural plasticity and consequently learning and memory outside the context of reproduction with the general consensus that an increase in circulating cytokines has detrimental effects on neurogenesis, long-term potentiation, and remodeling of neural circuits, as well as learning and memory (see [206] for review), they hypothesized that their observations were due to the increase in cytokine levels during pregnancy as described above. Indeed, pregnant nude mice lacking the entire T-cell population did only show a modest decrease in cell proliferation. However, if those mice were reconstituted with T-cells the pregnancy-induced decrease in hippocampal neurogenesis was restored [127]. Importantly, the altered cytokine levels during pregnancy seem to have wide-ranging consequences on hippocampal integrity. Cipolla et al. have nicely shown that treatment of hippocampal slices with serum from pregnant rats for 48 h caused morphological changes in microglia characteristics of activation and neuroinflammation of hippocampal neurons [207]. These are interesting findings, especially with respect to the fact that preeclampsia is a state of immune system hyperactivity (for review see [208]) and human studies showing severe impairments in memory function after preeclampsia [209]. Although there are no animal models of preeclampsia assessing hippocampal neurogenesis or microglia to date, it would be interesting to see if memory impairments after preeclamptic pregnancies are due to cytokine-induced alterations in hippocampal neurons.

The mechanism of the lactation-associated decrease in hippocampal cell proliferation was nicely elucidated by the group around Leuner. In a number of elegant studies in rats they revealed that the main drive for the reduction in cell proliferation on LD2 and LD8 is the hypercorticism observed during that time, which is directly linked to the presence of pups. Accordingly, lowering CORT levels by either pup removal or adrenalectomy prevented the decrease in hippocampal cell proliferation [131]. Similarly, chronic injection of high CORT (40 mg/kg/day) during gestation or the postpartum period leads to a decrease in cell proliferation in rats when compared to an oil-treated control group [210]. These results are in contrast to our recent findings in rats where we were able to show that chronic stress during lactation reverses the lactation-associated decrease in cell proliferation despite a basal increase in CORT levels on LD6 [61]. These results may initially seem paradoxical, as stress or high CORT levels are commonly thought to impair adult hippocampal neurogenesis [210–213]. However, it has been shown that the DG proliferation rate may habituate to stress exposure and, thus, display a reduced sensitivity to HPA axis hormones [211]. Moreover, despite the literature supporting a link between a reduction in adult hippocampal neurogenesis and high basal CORT levels during stress and lactation, only a small percentage of precursor cells express CORT receptors [214].

Aside from the role of glucocorticoids in regulating peripartum-associated hippocampal neurogenesis there are a few other pregnancy/lactation hormones that might contribute to the observed changes. In fact, estradiol, which increases 50-fold throughout pregnancy and drops during late pregnancy has been shown to decrease cell proliferation during early lactation in a hormone-stimulated pregnancy regimen in rats [132]. To date there is no proof that OXT would affect hippocampal neurogenesis during the peripartum period. However, given the fact that OXT levels are high during that time and OXT has been shown to promote hippocampal neurogenesis in male rats even under conditions of chronic stress [215], a potential regulatory involvement of this neuropeptide seems to be feasible. Therefore, further studies are highly desired to elucidate mechanisms underlying peripartum-associated alterations in hippocampal neurogenesis, particularly given the clear link between hippocampal neurogenesis, stress-related mental illnesses, and antidepressant treatment [216–220], and with regard to the fact that the peripartum period is a time of high susceptibility for women to develop mood or anxiety disorders (see [1] for review). Interestingly, we recently showed that chronic stress during lactation severely affects different stages of hippocampal neurogenesis [61]. Aside from the above-mentioned increase in hippocampal cell proliferation chronic stress exclusively reduced the number of newly generated neurons without affecting the astrocytic niche. This is particularly striking given the fact that the addition of new neurons is thought to be of importance in relation to maternal bonding and care [221, 222] and* vice versa *[126], which is severely affected not only in women with PPD [223], but also in rodents showing a change in maternal care and anxiety after chronic peripartum stress [151, 164, 224]. In this context, a dysregulation of the OXT system might be definitely taken into consideration as low plasma OXT concentrations during midpregnancy have been shown to significantly predict PPD symptoms two weeks postpartum in humans [225] and a reduced OXT mRNA expression in late pregnancy has been shown to be correlated with abnormal maternal behavior and anxiety in rodents [151].

Other factors that might contribute not only to peripartum-associated forms of neuroplasticity, but also with significant importance for proper placental development, are PlGF, VEGF, and their receptors, that are expressed in placental tissue and the brain (see [226, 227] for review). Although there is increasing evidence for a parallelism between vessel and nerve patterning in humans and rodents [228–230], there is a lack of research assessing the role of PlGF and VEGF in various forms of peripartum-associated neuroplasticity. This is astonishing, given the importance of a sufficient availability of these angiogenic factors in proper placentation as seen in humans [227, 231, 232] and the fact that both proteins have already been shown to be positively involved in neurogenic processes outside the context of pregnancy or lactation. In more detail, PlGF promotes the survival of primary cortical neurons in an* in vitro *model of permanent middle cerebral artery occlusion [233]. Similarly, VEGF stimulates the expansion of neural stem cells* in vitro *[234] and has been shown to be essential for the increase in hippocampal blood vessel density, neurogenesis in the OB and DG, and a resulting antidepressant effect in rats [234, 235]. Thus, it might be speculated that sufficient high levels of both PlGF and VEGF during the peripartum period are required to ensure neuroplasticity of the maternal brain and consequently maternal mental health. Although, to date there is no proof for an involvement of placental factors in the development of postpartum mood or anxiety disorders, it is clear that a malfunction in those systems mainly drives placental and endothelial dysfunction in preeclampsia. Characterized by the core symptoms hypertension and proteinuria after 20 weeks of gestation, preeclampsia is a leading cause of maternal and fetal morbidity and mortality [227]. Although there has been extensive research over the last decades revealing mechanisms underlying the pathology of preeclampsia, what is the cause and what is the consequence of the disease remain elusive. Nevertheless, one key mechanism seems to be based on a dysfunction of cytotrophoblasts, which, under normal conditions, acquire tumor-like properties that allow them to invade the uterus and promote vasculogenesis and subsequent angiogenesis by secreting VEGF and PlGF [236, 237]. In preeclamptic patients unbound plasma levels of these angiogenic factors have been shown to be severely diminished due to an increased expression of soluble fms-like tyrosine kinase sFlt-1 [238], which diminishes the binding of VEGF and PlGF to their usual transmembrane receptor Flt-1. This in fact will start up a couple of processes that will result in abnormal placental perfusion and microvascular oxidative damage (for review see [227]) and the occurrence of the typical core symptoms. Indeed, lowering the elevated level of sFlt-1 in very preterm preeclamptic women by dextran sulfate apheresis effectively improved the outcome for mother and fetus [239]. As mentioned above, other symptoms of preeclampsia, aside from proteinuria and hypertension, are severe cognitive impairments of the mothers during lactation [209]. Although the observed memory impairments might be due to an increased CNS permeability during preeclamptic pregnancy [240], the fact that PlGF and VEGF act as neuroprotective and neurotrophic as discussed above raises the question about a reduced neuroplasticity in preeclamptic women that might underlie the observed impairments in cognition.

## 6. Conclusions

To date there has been extensive research revealing peripartum-associated adaptations on the physiological and behavioral level, as well as on several forms of neuronal plasticity of the maternal brain. As outlined throughout the review there have been numerous studies nicely revealing that changes in the OXT and PRL system, altered levels of angiogenic factors like PlGF and VEGF, as well as immunological parameters tremendously contribute to adaptations of the maternal physiology, the function of “maternal” brain regions, and the resultant behavioral repertoire. Moreover, it is known that malfunction of these systems can lead to peripartum-associated physiological and psychological pathologies such as preeclampsia or depression. Although, there is an incontrovertible parallelism between factors regulating vasculogenesis and angiogenesis during placentation as well as neuroplasticity, there is a lack of research perceiving the placenta and the brain, as well as their regulatory mechanisms rather as a peripartum entity than as isolated organs. Thus, as VEGF and PlGF might play bigger roles than currently anticipated and the possibility that the cytokine and immune status of women during and after pregnancy might be involved in the physiological and structural changes observed in these critical periods of life, future studies are highly desirable to answer those open questions.

## Figures and Tables

**Table 1:** Examples of different forms of peripartum-associated neural plasticity in different relevant brain regions of mammals.

Brain region	Alteration	Time of occurrence	Species	Reference
Whole brain	↓Brain size	Pregnancy/Lactation	Human	[60]
	↓Brain weight	Lactation	Rat	[61]

Lateral ventricle	↓Volume	Pregnancy/Lactation	Human	[60]

Pituitary	↑Size	Pregnancy	Human	[62]
	↓Size	Lactation	Human, Rat	[63–68]

Hypothalamus				
SON	↑Volume	Lactation	Rat	[71–73]
	↑Axosomatic and axodendritic synapses on OXT neurons	Lactation	Rat	[98]
	↑Number of shared synapses on OXT neurons	Lactation	Rat	[71, 97, 100, 102]
	Altered excitatory and inhibitory input to OXT neurons	Lactation	Rat	[71, 98, 102, 105]
	↓Dendritic length and branching of OXT neurons	Lactation	Rat	[153]
PVN	Glial retraction of OXT neurons and dendrites	Lactation	Rat	[73, 101, 102]
	↑ICD	Lactation	Rat	[74]

Hippocampus	↓Volume	Lactation	Rat	[61]
	↓Cell proliferation	Lactation	Rat, Sheep	[61, 125, 130–132]
	↓Cell survival	Lactation	Rat	[131]
	↓Dendritic length and complexity of CA1/CA3 pyramidal neurons	Pregnancy/Lactation	Rat	[129, 137]
	↑Spines density on CA1 pyramidal neurons	Pregnancy/Lactation	Rat	[131, 140]

SVZ/OB	↑Cell proliferation SVZ	Pregnancy	Mouse	[115, 116]
	↑Number of interneurons OB	Lactation	Mouse	[115]
	↓Cell proliferation SVZ	Pregnancy/Lactation	Rat, Sheep	[117, 125]
	↑Density of presynapses and spine stability	Lactation	Mouse	[139]

mPFC	↑Dendritic length and spine density	Lactation	Rat	[57]

ICD: inner capillary diameter; mPFC: medial prefrontal cortex; OB: olfactory bulb; OXT: oxytocin; PVN: nucleus paraventricularis; SON: nucleus supraopticus; SVZ: subventricular zone.

## References

[1] Hillerer KM, Neumann ID, Slattery DA (2012). From stress to postpartum mood and anxiety disorders: how chronic peripartum stress can impair maternal adaptations. *Neuroendocrinology*.

[2] Neumann ID (2001). Alterations in behavioral and neuroendocrine stress coping strategies in pregnant, parturient and lactating rats. *Progress in Brain Research*.

[3] Russell JA, Douglas AJ, Ingram CD (2001). Brain preparations for maternity—adaptive changes in behavioral and neuroendocrine systems during pregnancy and lactation. An overview. *Progress in Brain Research*.

[4] Walker C-D, Trottier G, Rochford J, Lavallee D (1995). Dissociation between behavioral and hormonal responses to the forced swim stress in lactating rats. *Journal of Neuroendocrinology*.

[5] Sibai BM (2003). Diagnosis and management of gestational hypertension and preeclampsia. *Obstetrics and Gynecology*.

[6] Zhang J, Meikle S, Trumble A (2003). Severe maternal morbidity associated with hypertensive disorders in pregnancy in the United States. *Hypertension in Pregnancy*.

[7] Colberg SR, Castorino K, Jovanovic : L (2013). Prescribing physical activity to prevent and manage gestational diabetes. *World Journal of Diabetes*.

[8] Stein G, Marsh A, Morton J (1981). Mental symptoms, weight changes, and electrolyte excretion in the first postpartum week. *Journal of Psychosomatic Research*.

[9] Josefsson A, Berg G, Nordin C, Sydsjö G (2001). Prevalence of depressive symptoms in late pregnancy and postpartum. *Acta Obstetricia et Gynecologica Scandinavica*.

[10] O’Hara MW, Neunaber DJ, Zekoski EM (1984). Prospective study of postpartum depression: prevalence, course, and predictive factors. *Journal of Abnormal Psychology*.

[11] Andersson L, Sundström-Poromaa I, Wulff M, Åström M, Bixo M (2006). Depression and anxiety during pregnancy and six months postpartum: a follow-up study. *Acta Obstetricia et Gynecologica Scandinavica*.

[12] Heron J, O’Connor TG, Evans J, Golding J, Glover V (2004). The course of anxiety and depression through pregnancy and the postpartum in a community sample. *Journal of Affective Disorders*.

[13] Kendell RE, Chalmers JC, Platz C (1987). Epidemiology of puerperal psychoses. *British Journal of Psychiatry*.

[14] Cross JC, Werb Z, Fisher SJ (1994). Implantation and the placenta: key pieces of the development puzzle. *Science*.

[15] Kaufmann P, Bruns U, Leiser R (1985). The fetal vascularisation of term human placental villi. II. Intermediate and terminal villi. *Anatomy and Embryology*.

[16] Leiser R, Luckhardt M, Kaufmann P (1985). The fetal vascularisation of term human placental villi. I. Peripheral stem villi. *Anatomy and Embryology*.

[17] Lyall F (2005). Priming and remodelling of human placental bed spiral arteries during pregnancy—a review. *Placenta*.

[18] Moffett-King A (2002). Natural killer cells and pregnancy. *Nature Reviews Immunology*.

[19] Demir R, Seval Y, Huppertz B (2007). Vasculogenesis and angiogenesis in the early human placenta. *Acta Histochemica*.

[20] Zygmunt M, Herr F, Münstedt K, Lang U, Liang OD (2003). Angiogenesis and vasculogenesis in pregnancy. *European Journal of Obstetrics Gynecology and Reproductive Biology*.

[21] Park JE, Chen HH, Winer J, Houck KA, Ferrara N (1994). Placenta growth factor. Potentiation of vascular endothelial growth factor bioactivity, in vitro and in vivo, and high affinity binding to Flt-1 but not to Flk-1/KDR. *The Journal of Biological Chemistry*.

[22] Bowen JM, Chamley L, Keelan JA, Mitchell MD (2002). Cytokines of the placenta and extra-placental membranes: roles and regulation during human pregnancy and parturition. *Placenta*.

[23] Luppi P (2003). How immune mechanisms are affected by pregnancy. *Vaccine*.

[24] Altemus M, Deuster PA, Galliven E, Carter CS, Gold PW (1995). Suppression of hypothalmic-pituitary-adrenal axis responses to stress in lactating women. *Journal of Clinical Endocrinology and Metabolism*.

[25] Heinrichs M, Meinlschmidt G, Neumann I (2001). Effects of suckling on hypothalamic-pituitary-adrenal axis responses to psychosocial stress in postpartum lactating women. *Journal of Clinical Endocrinology and Metabolism*.

[26] Douglas AJ, Johnstone HA, Wigger A, Landgraf R, Russell JA, Neumann ID (1998). The role of endogenous opioids in neurohypophysial and hypothalamo- pituitary-adrenal axis hormone secretory responses to stress in pregnant rats. *Journal of Endocrinology*.

[27] Kammerer M, Adams D, von Castelberg B, Glover V (2002). Pregnant women become insensitive to cold stress. *BMC Pregnancy and Childbirth*.

[28] Toufexis DJ, Tesolin S, Huang N, Walker C-D (1999). Altered pituitary sensitivity to corticotropin-releasing factor and arginine vasopressin participates in the stress hyporesponsiveness of lactation in the rat. *Journal of Neuroendocrinology*.

[29] Wigger A, Lörscher P, Oehler I, Keck ME, Naruo T, Neumann ID (1999). Nonresponsiveness of the rat hypothalamo-pituitary-adrenocortical axis to parturition-related events: inhibitory action of endogenous opioids. *Endocrinology*.

[30] Brunton PJ, Russell JA (2008). The expectant brain: adapting for motherhood. *Nature Reviews Neuroscience*.

[31] Brunton PJ, Russell JA, Douglas AJ (2008). Adaptive responses of the maternal hypothalamic-pituitary-adrenal axis during pregnancy and lactation. *Journal of Neuroendocrinology*.

[32] Douglas AJ (2005). Central noradrenergic mechanisms underlying acute stress responses of the Hypothalamo-pituitary-adrenal axis: adaptations through pregnancy and lactation. *Stress*.

[33] Russell JA, Douglas AJ, Brunton PJ (2008). Reduced hypothalamo-pituitary-adrenal axis stress responses in late pregnancy: central opioid inhibition and noradrenergic mechanisms. *Annals of the New York Academy of Sciences*.

[34] Slattery DA, Neumann ID (2008). No stress please! Mechanisms of stress hyporesponsiveness of the maternal brain. *Journal of Physiology*.

[35] Neumann ID, Johnstone HA, Hatzinger M (1998). Attenuated neuroendocrine responses to emotional and physical stressors in pregnant rats involve adenohypophysial changes. *Journal of Physiology*.

[36] Shanks N, Windle RJ, Perks P, Wood S, Ingram CD, Lightman SL (1999). The hypothalamic-pituitary-adrenal axis response to endotoxin is attenuated during lactation. *Journal of Neuroendocrinology*.

[37] Walker C, Anand KJ, Plotsky PM, McEwen B (2001). Development of the hypothalamic-pituitary-adrenal axis and the stress response. *Coping With the Environment: Neural and Endocrine Mechanisms*.

[38] Windle RJ, Brady MM, Kunanandam T (1997). Reduced response of the hypothalamo-pituitary-adrenal axis to *α*1- agonist stimulation during lactation. *Endocrinology*.

[39] Figueira RJ, Peabody MF, Lonstein JS (2008). Oxytocin receptor activity in the ventrocaudal periaqueductal gray modulates anxiety-related behavior in postpartum rats. *Behavioral Neuroscience*.

[40] Grattan DR, Pi XJ, Andrews ZB (2001). Prolactin receptors in the brain during pregnancy and lactation: implications for behavior. *Hormones and Behavior*.

[41] Kokay IC, Bull PM, Davis RL, Ludwig M, Grattan DR (2006). Expression of the long form of the prolactin receptor in magnocellular oxytocin neurons is associated with specific prolactin regulation of oxytocin neurons. *American Journal of Physiology: Regulatory Integrative and Comparative Physiology*.

[42] Zingg HH, Rozen F, Breton C (1996). Gonadal steroid regulation of oxytocin and oxytocin receptor gene expression. *Advances in Experimental Medicine and Biology*.

[43] Neumann I, Ludwig M, Engelmann M, Pittman QJ, Landgraf R (1993). Simultaneous microdialysis in blood and brain: oxytocin and vasopressin release in response to central and peripheral osmotic stimulation and suckling in the rat. *Neuroendocrinology*.

[44] Neumann ID, Torner L, Wigger A (1999). Brain oxytocin: differential inhibition of neuroendocrine stress responses and anxiety-related behaviour in virgin, pregnant and lactating rats. *Neuroscience*.

[45] Neumann ID, Wigger A, Torner L, Holsboer F, Landgraf R (2000). Brain oxytocin inhibits basal and stress-induced activity of the hypothalamo-pituitary-adrenal axis in male and female rats: partial action within the paraventricular nucleus. *Journal of Neuroendocrinology*.

[46] Bosch OJ (2011). Maternal nurturing is dependent on her innate anxiety: the behavioral roles of brain oxytocin and vasopressin. *Hormones and Behavior*.

[47] Bridges R (2008). *Neurobiology of the Parental Brain*.

[48] Maestripieri D, Hoffman CL, Anderson GM, Carter CS, Higley JD (2009). Mother-infant interactions in free-ranging rhesus macaques: relationships between physiological and behavioral variables. *Physiology and Behavior*.

[49] Pedersen CA, Prange AJ (1985). Oxytocin and mothering behavior in the rat. *Pharmacology and Therapeutics*.

[50] Carter CS, Altemus M (1997). Integrative functions of lactational hormones in social behavior and stress management. *Annals of the New York Academy of Sciences*.

[51] Grattan DR (2001). The actions of prolactin in the brain during pregnancy and lactation. *Progress in Brain Research*.

[52] Keller-Wood M (1995). Reflex regulation of hormonal responses during pregnancy. *Clinical and Experimental Pharmacology and Physiology*.

[53] Lightman SL, Windle RJ, Wood SA, Kershaw YM, Shanks N, Ingram CD (2001). Peripartum plasticity within the hypothalamo-pituitary-adrenal axis. *Progress in Brain Research*.

[54] Stern JM, Goldman L, Levine S (1973). Pituitary adrenal responsiveness during lactation in rats. *Neuroendocrinology*.

[55] Numan M (2007). Motivational systems and the neural circuitry of maternal behavior in the rat. *Developmental Psychobiology*.

[56] Neumann ID (2008). Brain oxytocin: a key regulator of emotional and social behaviours in both females and males. *Journal of Neuroendocrinology*.

[57] Leuner B, Gould E (2010). Dendritic growth in medial prefrontal cortex and cognitive flexibility are enhanced during the postpartum period. *The Journal of Neuroscience*.

[58] Euston DR, Gruber AJ, McNaughton : BL (2012). The role of medial prefrontal cortex in memory and decision making. *Neuron*.

[59] Koehl M, Abrous DN (2011). A new chapter in the field of memory: adult hippocampal neurogenesis. *European Journal of Neuroscience*.

[60] Oatridge A, Holdcroft A, Saeed N (2002). Change in brain size during and after pregnancy: study in healthy women and women with preeclampsia. *American Journal of Neuroradiology*.

[61] Hillerer KM, Neumann ID, Couillard-Despres S, Aigner L, Slattery : DA (2014). Lactation-induced reduction in hippocampal neurogenesis is reversed by repeated stress exposure. *Hippocampus*.

[62] Asa SL, Penz G, Kovacs K, Ezrin C (1982). Prolactin cells in the human pituitary. A quantitative immunocytochemical analysis. *Archives of Pathology and Laboratory Medicine*.

[63] Dinç H, Esen F, Demirci A, Sari A, Resit Gümele H (1998). Pituitary dimensions and volume measurements in pregnancy and post partum: MR assessment. *Acta Radiologica*.

[64] Elster AD, Sanders TG, Vines FS, Chen MYM (1991). Size and shape of the pituitary gland during pregnancy and post partum: measurement with MR imaging. *Radiology*.

[65] Miki Y, Kataoka ML, Shibata T (2005). The pituitary gland: changes on MR images during the 1st year after delivery. *Radiology*.

[66] Farquhar MG (1961). Origin and fate of secretory granules in cells of the anterior pituitary gland. *Transactions of the New York Academy of Sciences*.

[67] Haggi ES, Torres AI, Maldonado CA, Aoki A (1986). Regression of redundant lactotrophs in rat pituitary gland after cessation of lactation. *Journal of Endocrinology*.

[68] Smith RE, Farquhar MG (1966). Lysosome function in the regulation of the secretory process in cells of the anterior pituitary gland. *The Journal of Cell Biology*.

[69] Scheithauer BW, Sano T, Kovacs KT, Young WF, Ryan N, Randall RV (1990). The pituitary gland in pregnancy: a clinicopathologic and immunohistochemical study of 69 cases. *Mayo Clinic Proceedings*.

[70] Keyser-Marcus L, Stafisso-Sandoz G, Gerecke K (2001). Alterations of medial preoptic area neurons following pregnancy and pregnancy-like steroidal treatment in the rat. *Brain Research Bulletin*.

[71] El Majdoubi M, Poulain DA, Theodosis DT (1997). Lactation-induced plasticity in the supraoptic nucleus augments axodendritic and axosomatic GABAergic and glutamatergic synapses: an ultrastructural analysis using the disector method. *Neuroscience*.

[72] Salm AK, Modney BK, Hatton GI (1988). Alterations in supraoptic nucleus ultrastructure of maternally behaving virgin rats. *Brain Research Bulletin*.

[73] Theodosis DT, Chapman DB, Montagnese C (1986). Structural plasticity in the hypothalamic supraoptic nucleus at lactation affects oxytocin-, but not vasopressin-secreting neurones. *Neuroscience*.

[74] Cortes-Sol A, Lara-Garcia M, Alvarado M, Hudson R, Berbel P, Pacheco P (2013). Inner capillary diameter of hypothalamic paraventricular nucleus of female rat increases during lactation. *BMC Neuroscience*.

[75] Sugawara J, Mitsui-Saito M, Hoshiai T, Hayashi C, Kimura Y, Okamura K (2005). Circulating endothelial progenitor cells during human pregnancy. *Journal of Clinical Endocrinology and Metabolism*.

[76] Lin C, Rajakumar A, Plymire DA, Verma V, Markovic N, Hubel CA (2009). Maternal endothelial progenitor colony-forming units with macrophage characteristics are reduced in preeclampsia. *American Journal of Hypertension*.

[77] Sugawara J, Mitsui-Saito M, Hayashi C (2005). Decrease and senescence of endothelial progenitor cells in patients with preeclampsia. *Journal of Clinical Endocrinology and Metabolism*.

[78] Risberg A, Olsson K, Lyrenäs S, Sjöquist M (2009). Plasma vasopressin, oxytocin, estradiol, and progesterone related to water and sodium excretion in normal pregnancy and gestational hypertension. *Acta Obstetricia et Gynecologica Scandinavica*.

[79] Rosso L, Peteri-Brunbäck B, Poujeol P, Hussy N, Mienville J-M (2004). Vasopressin-induced taurine efflux from rat pituicytes: a potential negative feedback for hormone secretion. *Journal of Physiology*.

[80] Champagne FA, Weaver ICG, Diorio J, Dymov S, Szyf M, Meaney MJ (2006). Maternal care associated with methylation of the estrogen receptor-*α*1b promoter and estrogen receptor-*α* expression in the medial preoptic area of female offspring. *Endocrinology*.

[81] Champagne FA, Weaver ICG, Diorio J, Sharma S, Meaney MJ (2003). Natural variations in maternal care are associated with estrogen receptor *α* expression and estrogen sensitivity in the medial preoptic area. *Endocrinology*.

[82] Byrnes EM, Babb JA, Bridges RS (2009). Differential expression of oestrogen receptor *α* following reproductive experience in young and middle-aged female rats. *Journal of Neuroendocrinology*.

[83] Ehret G, Buckenmaier J (1994). Estrogen-receptor occurrence in the female mouse brain: effects of maternal experience, ovariectomy, estrogen and anosmia. *Journal of Physiology Paris*.

[84] Meurisse M, Gonzalez A, Delsol G, Caba M, Lévy F, Poindron P (2005). Estradiol receptor-*α* expression in hypothalamic and limbic regions of ewes is influenced by physiological state and maternal experience. *Hormones and Behavior*.

[85] Poindron P, Orgeur P, Le Neindre P (1980). Influence of the blood concentration of prolactin on the length of the sensitive period for establishing maternal behavior in sheep at parturition. *Hormones and Behavior*.

[86] Bale TL, Pedersen CA, Dorsa DM (1996). CNS oxytocin receptor mRNA expression and regulation by gonadal steroids. *Advances in Experimental Medicine and Biology*.

[87] Champagne F, Diorio J, Sharma S, Meaney MJ (2001). Naturally occurring variations in maternal behavior in the rat are associated with differences in estrogen-inducible central oxytocin receptors. *Proceedings of the National Academy of Sciences of the United States of America*.

[88] Champagne FA, Meaney MJ (2006). Stress during gestation alters postpartum maternal care and the development of the offspring in a rodent model. *Biological Psychiatry*.

[89] Mann PE, Bridges RS (2002). Prolactin receptor gene expression in the forebrain of pregnant and lactating rats. *Molecular Brain Research*.

[90] Pi XJ, Grattan DR (1999). Increased expression of both short and long forms of prolactin receptor mRNA in hypothalamic nuclei of lactating rats. *Journal of Molecular Endocrinology*.

[91] Torner L, Toschi N, Nava G, Clapp C, Neumann ID (2002). Increased hypothalamic expression of prolactin in lactation: involvement in behavioural and neuroendocrine stress responses. *European Journal of Neuroscience*.

[92] Torner L, Toschi N, Pohlinger A, Landgraf R, Neumann ID (2001). Anxiolytic and anti-stress effects of brain prolactin: improved efficacy of antisense targeting of the prolactin receptor by molecular modeling. *The Journal of Neuroscience*.

[93] Meaney MJ, Viau V, Aitken DH, Bhatnagar S (1989). Glucocorticoid receptors in brain and pituitary of the lactating rat. *Physiology and Behavior*.

[94] Caughey SD, Klampfl SM, Bishop VR (2011). Changes in the intensity of maternal aggression and central oxytocin and vasopressin V1a receptors across the peripartum period in the rat. *Journal of Neuroendocrinology*.

[95] Bosch OJ, Pförtsch J, Beiderbeck DI, Landgraf R, Neumann ID (2010). Maternal behaviour is associated with vasopressin release in the medial preoptic area and bed nucleus of the stria terminalis in the rat. *Journal of Neuroendocrinology*.

[96] Debanne D, Daoudal G, Sourdet V, Russier M (2003). Brain plasticity and ion channels. *Journal of Physiology Paris*.

[97] Theodosis DT, Poulain DA, Vincent J-D (1981). Possible morphological bases for synchronisation of neuronal firing in the rat supraoptic nucleus during lactation. *Neuroscience*.

[98] Gies U, Theodosis DT (1994). Synaptic plasticity in the rat supraoptic nucleus during lactation involves GABA innervation and oxytocin neurons: a quantitative immunocytochemical analysis. *The Journal of Neuroscience*.

[99] Montagnese CM, Poulain DA, Vincent J-D, Theodosis DT (1987). Structural plasticity in the rat supraoptic nucleus during gestation, post-partum lactation and suckling-induced pseudogestation and lactation. *Journal of Endocrinology*.

[100] Theodosis DT, Poulain DA (1984). Evidence for structural plasticity in the supraoptic nucleus of the rat hypothalamus in relation to gestation and lactation. *Neuroscience*.

[101] Chapman DB, Theodosis DT, Montagnese C (1986). Osmotic stimulation causes structural plasticity of neurone-glia relationships of the oxytocin but not vasopressin secreting neurones in the hypothalamic supraoptic nucleus. *Neuroscience*.

[102] Majdoubi MEI, Poulain DA, Theodosis DT (1996). The glutamatergic innervation of oxytocin- and vasopressin-secreting neurons in the rat supraoptic nucleus and its contribution to lactation-induced synaptic plasticity. *European Journal of Neuroscience*.

[103] Volterra A, Meldolesi J (2005). Astrocytes, from brain glue to communication elements: the revolution continues. *Nature Reviews Neuroscience*.

[104] Langle SL, Poulain DA, Theodosis DT (2003). Induction of rapid, activity-dependent neuronal-glial remodelling in the adult rat hypothalamus in vitro. *European Journal of Neuroscience*.

[105] Michaloudi HC, El Majdoubi M, Poulain DA, Papadopoulos GC, Theodosis DT (1997). The noradrenergic innervation of identified hypothalamic magnocellular somata and its contribution to lactation-induced synaptic plasticity. *Journal of Neuroendocrinology*.

[106] Jourdain P, Dupouy B, Bonhomme R, Poulain DA, Israel JM, Theodosis DT (1999). Visualization of local afferent inputs to magnocellular oxytocin neurons in vitro. *European Journal of Neuroscience*.

[107] Roland BL, Sawchenko PE (1993). Local origins of some GABAergic projections to the paraventricular and supraoptic nuclei of the hypothalamus in the rat. *The Journal of Comparative Neurology*.

[108] Boudaba C, Szabó K, Tasker JG (1996). Physiological mapping of local inhibitory inputs to the hypothalamic paraventricular nucleus. *The Journal of Neuroscience*.

[109] Zaborszky L, Leranth Cs. LC, Makara GB, Palkovits M (1975). Quantitative studies on the supraoptic nucleus in the rat. II. Afferent fiber connections. *Experimental Brain Research*.

[110] Poulain DA, Wakerley JB (1982). Electrophysiology of hypothalamic magnocellular neurones secreting oxytocin and vasopressin. *Neuroscience*.

[111] Theodosis DT, Poulain DA (2001). Maternity leads to morphological synaptic plasticity in the oxytocin system. *Progress in Brain Research*.

[112] Lightman SL, Young WS (1989). Lactation inhibits stress-mediated secretion of corticosterone and oxytocin and hypothalamic accumulation of corticotropin-releasing factor and enkephalin messenger ribonucleic acids. *Endocrinology*.

[113] Abrous DN, Koehl M, Le Moal M (2005). Adult neurogenesis: from precursors to network and physiology. *Physiological Reviews*.

[114] Lledo P-M, Alonso M, Grubb MS (2006). Adult neurogenesis and functional plasticity in neuronal circuits. *Nature Reviews Neuroscience*.

[115] Shingo T, Gregg C, Enwere E (2003). Pregnancy-stimulated neurogenesis in the adult female forebrain mediated by prolactin. *Science*.

[116] Larsen CM, Grattan DR (2010). Prolactin-induced mitogenesis in the subventricular zone of the maternal brain during early pregnancy is essential for normal postpartum behavioral responses in the mother. *Endocrinology*.

[117] Furuta M, Bridges RS (2005). Gestation-induced cell proliferation in the rat brain. *Developmental Brain Research*.

[118] Taupin P (2007). BrdU immunohistochemistry for studying adult neurogenesis: paradigms, pitfalls, limitations, and validation. *Brain Research Reviews*.

[119] Sakamoto M, Imayoshi I, Ohtsuka T, Yamaguchi M, Mori K, Kageyama R (2011). Continuous neurogenesis in the adult forebrain is required for innate olfactory responses. *Proceedings of the National Academy of Sciences of the United States of America*.

[120] Feierstein CE, Lazarini F, Wagner S (2010). Disruption of adult neurogenesis in the olfactory bulb affects social interaction but not maternal behavior. *Frontiers in Behavioral Neuroscience*.

[121] Magavi SSP, Mitchell BD, Szentirmai O, Carter BS, Macklis JD (2005). Adult-born and preexisting olfactory granule neurons undergo distinct experience-dependent modifications of their olfactory responses in vivo. *The Journal of Neuroscience*.

[122] Schaal B, LeCamus J, Campons R (1986). Presumed olfactory exchanges between mother and neonate in humans. *Ethologie et Psychologie de L'Enfant*.

[123] Brennan PA, Keverne EB (1997). Neural mechanisms of mammalian olfactory learning. *Progress in Neurobiology*.

[124] Kendrick KM, Da Costa APC, Broad KD (1997). Neural control of maternal behaviour and olfactory recognition of offspring. *Brain Research Bulletin*.

[125] Brus M, Meurisse M, Franceschini I, Keller M, Lévy F (2010). Evidence for cell proliferation in the sheep brain and its down-regulation by parturition and interactions with the young. *Hormones and Behavior*.

[126] Furuta M, Bridges RS (2009). Effects of maternal behavior induction and pup exposure on neurogenesis in adult, virgin female rats. *Brain Research Bulletin*.

[127] Rolls A, Schori H, London A, Schwartz M (2008). Decrease in hippocampal neurogenesis during pregnancy: a link to immunity. *Molecular Psychiatry*.

[128] Banasr M, Hery M, Brezun JM, Daszuta A (2001). Serotonin mediates oestrogen stimulation of cell proliferation in the adult dentate gyrus. *European Journal of Neuroscience*.

[129] Pawluski JL, Valenca A, Santos AI, Costa-Nunes JP, Steinbusch HW, Strekalova : T (2010). Pregnancy or stress decrease complexity of ca3 pyramidal neurons in the hippocampus of adult female rats. *Neuroscience*.

[130] Darnaudéry M, Perez-Martin M, Del Favero F, Gomez-Roldan C, Garcia-Segura LM, Maccari S (2007). Early motherhood in rats is associated with a modification of hippocampal function. *Psychoneuroendocrinology*.

[131] Leuner B, Mirescu C, Noiman L, Gould E (2007). Maternal experience inhibits the production of immature neurons in the hippocampus during the postpartum period through elevations in adrenal steroids. *Hippocampus*.

[132] Pawluski JL, Galea LAM (2007). Reproductive experience alters hippocampal neurogenesis during the postpartum period in the dam. *Neuroscience*.

[133] Glasper ER, Kozorovitskiy Y, Pavlic A, Gould E (2011). Paternal experience suppresses adult neurogenesis without altering hippocampal function in Peromyscus californicus. *The Journal of Comparative Neurology*.

[134] Mak GK, Weiss S (2010). Paternal recognition of adult offspring mediated by newly generated CNS neurons. *Nature Neuroscience*.

[135] Ruscio MG, Sweeny TD, Hazelton JL, Suppatkul P, Boothe E, Carter CS (2008). Pup exposure elicits hippocampal cell proliferation in the prairie vole. *Behavioural Brain Research*.

[136] Leuner B, Gould E (2010). Structural plasticity and hippocampal function. *Annual Review of Psychology*.

[137] Pawluski JL, Galea LAM (2006). Hippocampal morphology is differentially affected by reproductive experience in the mother. *Journal of Neurobiology*.

[138] Magariños AM, McEwen BS (1995). Stress-induced atrophy of apical dendrites of hippocampal CA3c neurons: involvement of glucocorticoid secretion and excitatory amino acid receptors. *Neuroscience*.

[139] Kopel H, Schechtman E, Groysman M, Mizrahi : A (2012). Enhanced synaptic integration of adult-born neurons in the olfactory bulb of lactating mothers. *The Journal of Neuroscience*.

[140] Kinsley CH, Trainer R, Stafisso-Sandoz G (2006). Motherhood and the hormones of pregnancy modify concentrations of hippocampal neuronal dendritic spines. *Hormones and Behavior*.

[141] Woolley CS, Gould E, Frankfurt M, McEwen BS (1990). Naturally occurring fluctuation in dendritic spine density on adult hippocampal pyramidal neurons. *The Journal of Neuroscience*.

[142] Kozorovitskiy Y, Gross CG, Kopil C (2005). Experience induces structural and biochemical changes in the adult primate brain. *Proceedings of the National Academy of Sciences of the United States of America*.

[143] Brusco J, Wittmann R, de Azevedo MS (2008). Plasma hormonal profiles and dendritic spine density and morphology in the hippocampal CA1 stratum radiatum, evidenced by light microscopy, of virgin and postpartum female rats. *Neuroscience Letters*.

[144] Fox MT, Barense MD, Baxter MG (2003). Perceptual attentional set-shifting is impaired in rats with neurotoxic lesions of posterior parietal cortex. *The Journal of Neuroscience*.

[145] Liston C, Miller MM, Goldwater DS (2006). Stress-induced alterations in prefrontal cortical dendritic morphology predict selective impairments in perceptual attentional set-shifting. *The Journal of Neuroscience*.

[146] Radley JJ, Rocher AB, Miller M (2006). Repeated stress induces dendritic spine loss in the rat medial prefrontal cortex. *Cerebral Cortex*.

[147] Workman JL, Brummelte S, Galea : LA (2013). Postpartum corticosterone administration reduces dendritic complexity and increases the density of mushroom spines of hippocampal ca3 arbours in dams. *Journal of Neuroendocrinology*.

[148] Febo M, Numan M, Ferris CF (2005). Functional magnetic resonance imaging shows oxytocin activates brain regions associated with mother-pup bonding during suckling. *The Journal of Neuroscience*.

[149] Fleming AS, Korsmit M (1996). Plasticity in the maternal circuit: effects of maternal experience on Fos-lir in hypothalamic, limbic, and cortical structures in the postpartum rat. *Behavioral Neuroscience*.

[150] Neumann ID (2007). Stimuli and consequences of dendritic release of oxytocin within the brain. *Biochemical Society Transactions*.

[151] Hillerer KM, Reber SO, Neumann ID, Slattery DA (2011). Exposure to chronic pregnancy stress reverses peripartum-associated adaptations: implications for postpartum anxiety and mood disorders. *Endocrinology*.

[152] Miller MM, Morrison JH, McEwen BS (2012). Basal anxiety-like behavior predicts differences in dendritic morphology in the medial prefrontal cortex in two strains of rats. *Behavioural Brain Research*.

[153] Stern JE, Armstrong WE (1998). Reorganization of the dendritic trees of oxytocin and vasopressin neurons of the rat supraoptic nucleus during lactation. *The Journal of Neuroscience*.

[154] Rall W (1977). *Core Conductor Theory and Cable Properties of Neurons*.

[155] Wakerley JB, Lincoln DW (1973). The milk ejection reflex of the rat: a 20 to 40 fold acceleration in the firing of paraventricular neurones during oxytocin release. *Journal of Endocrinology*.

[156] Higuchi T, Honda K, Takano S, Negoro H (1988). Reduced oxytocin response to osmotic stimulus and immobilization stress in lactating rats. *Journal of Endocrinology*.

[157] Koehler EM, McLemore GL, Tang W, Summy-Long JY (1993). Osmoregulation of the magnocellular system during pregnancy and lactation. *American Journal of Physiology: Regulatory Integrative and Comparative Physiology*.

[158] van Leengoed E, Kerker E, Swanson HH (1987). Inhibition of post-partum maternal behaviour in the rat by injecting an oxytocin antagonist into the cerebral ventricles. *Journal of Endocrinology*.

[159] Bridges RS, Robertson MC, Shiu RPC, Sturgis JD, Henriquez BM, Mann PE (1997). Central lactogenic regulation of maternal behavior in rats: steroid dependence, hormone specificity, and behavioral potencies of rat prolactin and rat placental lactogen I. *Endocrinology*.

[160] Bosch OJ, Neumann ID (2010). Vasopressin released within the central amygdala promotes maternal aggression. *European Journal of Neuroscience*.

[161] Insel TR (1990). Regional changes in brain oxytocin receptors post-partum: time-course and relationship to maternal behaviour. *Journal of Neuroendocrinology*.

[162] Popeski N, Amir S, Diorio J, Woodside B (2003). Prolactin and oxytocin interaction in the paraventricular and supraoptic nuclei: effects on oxytocin mRNA and nitric oxide synthase. *Journal of Neuroendocrinology*.

[163] Bosch OJ, Meddle SL, Beiderbeck DI, Douglas AJ, Neumann ID (2005). Brain oxytocin correlates with maternal aggression: link to anxiety. *The Journal of Neuroscience*.

[164] Maestripieri D, D’Amato FR (1991). Anxiety and maternal aggression in house mice (Mus musculus): a look at interindividual variability. *Journal of Comparative Psychology*.

[165] Parmigiani S, Palanza P, Rodgers J, Ferrari PF (1999). Selection, evolution of behavior and animal models in behavioral neuroscience. *Neuroscience and Biobehavioral Reviews*.

[166] Gammie SC, Negron A, Newman SM, Rhodes JS (2004). Corticotropin-releasing factor inhibits maternal aggression in mice. *Behavioral Neuroscience*.

[167] Gammie SC, Seasholtz AF, Stevenson SA (2008). Deletion of corticotropin-releasing factor binding protein selectively impairs maternal, but not intermale aggression. *Neuroscience*.

[168] Erskine MS, Denenberg VH, Goldman BD (1978). Aggression in the lactating rat: effects of intruder age and test arena. *Behavioral Biology*.

[169] Hard E, Hansen S (1985). Reduced fearfulness in the lactating rat. *Physiology and Behavior*.

[170] Galea LA, Wainwright SR, Roes MM, Duarte-Guterman P, Chow C, Hamson DK (2013). Sex, hormones and neurogenesis in the hippocampus: hormonal modulation of neurogenesis and potential functional implications. *Journal of Neuroendocrinology*.

[171] Macbeth AH, Luine VN (2010). Changes in anxiety and cognition due to reproductive experience: a review of data from rodent and human mothers. *Neuroscience and Biobehavioral Reviews*.

[172] Bodensteiner KJ, Cain P, Ray AS, Hamula LA (2006). Effects of pregnancy on spatial cognition in female Hooded Long-Evans rats. *Hormones and Behavior*.

[173] Galea LAM, Ormerod BK, Sampath S, Kostaras X, Wilkie DM, Phelps MT (2000). Spatial working memory and hippocampal size across pregnancy in rats. *Hormones and Behavior*.

[174] Kinsley CH, Madonia L, Gifford GW (1999). Motherhood improves learning and memory. *Nature*.

[175] Macbeth AH, Gautreaux C, Luine VN (2008). Pregnant rats show enhanced spatial memory, decreased anxiety, and altered levels of monoaminergic neurotransmitters. *Brain Research*.

[176] Paris JJ, Frye CA (2008). Estrous cycle, pregnancy, and parity enhance performance of rats in object recognition or object placement tasks. *Reproduction*.

[177] Galen Buckwalter J, Stanczyk FZ, McCleary CA (1999). Pregnancy, the postpartum, and steroid hormones: effects on cognition and mood. *Psychoneuroendocrinology*.

[178] de Groot RHM, Vuurman EFPM, Hornstra G, Jolles J (2006). Differences in cognitive performance during pregnancy and early motherhood. *Psychological Medicine*.

[179] Eidelman AI, Hoffmann NW, Kaitz M (1993). Cognitive deficits in women after childbirth. *Obstetrics and Gynecology*.

[180] Glynn LM (2010). Giving birth to a new brain: hormone exposures of pregnancy influence human memory. *Psychoneuroendocrinology*.

[181] Silber M, Almkvist O, Larsson B, Uvnas-Moberg K (1990). Temporary peripartal impairment in memory and attention and its possible relation to oxytocin concentration. *Life Sciences*.

[182] Vanston CM, Watson NV (2005). Selective and persistent effect of foetal sex on cognition in pregnant women. *NeuroReport*.

[183] Crawley RA, Dennison K, Carter C (2003). Cognition in pregnancy and the first year post-partum. *Psychology and Psychotherapy: Theory, Research and Practice*.

[184] Brindle PM, Brown MW, Brown J, Griffith HB, Turner GM (1991). Objective and subjective memory impairment in pregnancy. *Psychological Medicine*.

[185] Sharp K, Brindle PM, Brown MW, Turner GM (1993). Memory loss during pregnancy. *British Journal of Obstetrics and Gynaecology*.

[186] Lambert KG, Berry AE, Griffin G (2005). Pup exposure differentially enhances foraging ability in primiparous and nulliparous rats. *Physiology and Behavior*.

[187] Lemaire V, Billard J-M, Dutar P (2006). Motherhood-induced memory improvement persists across lifespan in rats but is abolished by a gestational stress. *European Journal of Neuroscience*.

[188] Pawluski JL, Vanderbyl BL, Ragan K, Galea LAM (2006). First reproductive experience persistently affects spatial reference and working memory in the mother and these effects are not due to pregnancy or ’mothering’ alone. *Behavioural Brain Research*.

[189] Pawluski JL, Walker SK, Galea LAM (2006). Reproductive experience differentially affects spatial reference and working memory performance in the mother. *Hormones and Behavior*.

[190] Cost KT, Lobell TD, Williams-Yee ZN, Henderson S, Dohanich : G (2013). The effects of pregnancy, lactation, and primiparity on object-in-place memory of female rats. *Hormones and Behavior*.

[191] Buckwalter JG, Buckwalter DK, Bluestein BW, Stanczyk FZ (2001). Pregnancy and post partum: changes in cognition and mood. *Progress in Brain Research*.

[192] Maeng LY, Shors TJ (2012). Once a mother, always a mother: maternal experience protects females from the negative effects of stress on learning. *Behavioral Neuroscience*.

[193] Epp JR, Chow C, Galea LA (2013). Hippocampus-dependent learning influences hippocampal neurogenesis. *Frontiers in Neuroscience*.

[194] Winocur G, Wojtowicz JM, Sekeres M, Snyder JS, Wang S (2006). Inhibition of neurogenesis interferes with hippocampus-dependent memory function. *Hippocampus*.

[195] Gatewood JD, Morgan MD, Eaton M (2005). Motherhood mitigates aging-related decrements in learning and memory and positively affects brain aging in the rat. *Brain Research Bulletin*.

[196] Macbeth AH, Scharfman HE, MacLusky NJ, Gautreaux C, Luine VN (2008). Effects of multiparity on recognition memory, monoaminergic neurotransmitters, and brain-derived neurotrophic factor (BDNF). *Hormones and Behavior*.

[197] Colucci M, Cammarata S, Assini A (2006). The number of pregnancies is a risk factor for Alzheimer’s disease. *European Journal of Neurology*.

[198] Theodosis DT, Bonhomme R, Vitiello S, Rougon G, Poulain DA (1999). Cell surface expression of polysialic acid on NCAM is a prerequisite for activity-dependent morphological neuronal and glial plasticity. *The Journal of Neuroscience*.

[199] Theodosis DT, Poulain DA (2000). Contribution of astrocytes to activity-dependent structural plasticity in the adult brain. *Advances in Experimental Medicine and Biology*.

[200] Edelman GM, Crossin KL (1991). Cell adhesion molecules: implications for a molecular histology. *Annual Review of Biochemistry*.

[201] Rutishauser U, Landmesser L (1996). Polysialic acid in the vertebrate nervous system: a promoter of plasticity in cell-cell interactions. *Trends in Neurosciences*.

[202] Montagnese C, Poulain DA, Theodosis DT (1990). Influence of ovarian steroids on the ultrastructural plasticity of the adult rat supraoptic nucleus induced by central administration of oxytocin. *Journal of Neuroendocrinology*.

[203] Theodosis DT, Montagnese C, Rodriguez F (1986). Oxytocin induces morphological plasticity in the adult hypothalamo-neurohypophysial system. *Nature*.

[204] Bonfanti L (1994). Expression of polysialylated neural cell adhesion molecule by proliferating cells in the subependymal layer of the adult rat, in its rostral extension and in the olfactory bulb. *Neuroscience*.

[205] Chazal G, Durbec P, Jankovski A, Rougon G, Cremer H (2000). Consequences of neural cell adhesion molecule deficiency on cell migration in the rostral migratory stream of the mouse. *The Journal of Neuroscience*.

[206] Yirmiya R, Goshen I (2011). Immune modulation of learning, memory, neural plasticity and neurogenesis. *Brain, Behavior, and Immunity*.

[207] Cipolla MJ, Pusic AD, Grinberg YY, Chapman AC, Poynter ME, Kraig RP (2012). Pregnant serum induces neuroinflammation and seizure activity via TNF*α*. *Experimental Neurology*.

[208] Ahn H, Park J, Gilman-Sachs A, Kwak-Kim J (2011). Immunologic Characteristics of Preeclampsia, a Comprehensive Review. *American Journal of Reproductive Immunology*.

[209] Brussé I, Duvekot J, Jongerling J, Steegers E, de Koning I (2008). Impaired maternal cognitive functioning after pregnancies complicated by severe pre-eclampsia: a pilot case-control study. *Acta Obstetricia et Gynecologica Scandinavica*.

[210] Brummelte S, Galea LAM (2010). Chronic high corticosterone reduces neurogenesis in the dentate gyrus of adult male and female rats. *Neuroscience*.

[211] Czéh B, Welt T, Fischer AK (2002). Chronic psychosocial stress and concomitant repetitive transcranial magnetic stimulation: effects on stress hormone levels and adult hippocampal neurogenesis. *Biological Psychiatry*.

[212] Pham K, Nacher J, Hof PR, McEwen BS (2003). Repeated restraint stress suppresses neurogenesis and induces biphasic PSA-NCAM expression in the adult rat dentate gyrus. *European Journal of Neuroscience*.

[213] Tanapat P, Hastings NB, Rydel TA, Galea LAM, Gould E (2001). Exposure to fox odor inhibits cell proliferation in the hippocampus of adult rats via an adrenal hormone-dependent mechanism. *The Journal of Comparative Neurology*.

[214] Cameron HA, Woolley CS, Gould E (1993). Adrenal steroid receptor immunoreactivity in cells born in the adult rat dentate gyrus. *Brain Research*.

[215] Leuner B, Caponiti JM, Gould E (2012). Oxytocin stimulates adult neurogenesis even under conditions of stress and elevated glucocorticoids. *Hippocampus*.

[216] Czéh B, Michaelis T, Watanabe T (2001). Stress-induced changes in cerebral metabolites, hippocampal volume, and cell proliferation are prevented by antidepressant treatment with tianeptine. *Proceedings of the National Academy of Sciences of the United States of America*.

[217] Dagyt G, Crescente I, Postema F (2011). Agomelatine reverses the decrease in hippocampal cell survival induced by chronic mild stress. *Behavioural Brain Research*.

[218] Revest J-M, Dupret D, Koehl M (2009). Adult hippocampal neurogenesis is involved in anxiety-related behaviors. *Molecular Psychiatry*.

[219] Santarelli L, Saxe M, Gross C (2003). Requirement of hippocampal neurogenesis for the behavioral effects of antidepressants. *Science*.

[220] Snyder JS, Soumier A, Brewer M, Pickel J, Cameron HA (2011). Adult hippocampal neurogenesis buffers stress responses and depressive behaviour. *Nature*.

[221] Gandelman R, Zarrow MX, Denenberg VH, Myers M (1971). Olfactory bulb removal eliminates maternal behavior in the mouse. *Science*.

[222] Leuner B, Shors TJ (2006). Learning during motherhood: a resistance to stress. *Hormones and Behavior*.

[223] Beck CT, Indman P (2005). The many faces of postpartum depression. *Journal of Obstetric, Gynecologic, and Neonatal Nursing*.

[224] Purcell RH, Sun B, Pass LL, Power ML, Moran TH, Tamashiro KLK (2011). Maternal stress and high-fat diet effect on maternal behavior, milk composition, and pup ingestive behavior. *Physiology and Behavior*.

[225] Skrundz M, Bolten M, Nast I, Hellhammer DH, Meinlschmidt G (2011). Plasma oxytocin concentration during pregnancy is associated with development of postpartum depression. *Neuropsychopharmacology*.

[226] Chaballe L, Schoenen J, Franzen R (2011). Placental growth factor: a tissue modelling factor with therapeutic potentials in neurology?. *Acta Neurologica Belgica*.

[227] Noris M, Perico N, Remuzzi G (2005). Mechanisms of disease: pre-eclampsia. *Nature Clinical Practice*.

[228] Autiero M, de Smet F, Claes F, Carmeliet P (2005). Role of neural guidance signals in blood vessel navigation. *Cardiovascular Research*.

[229] Carmeliet P (2003). Blood vessels and nerves: common signals, pathways and diseases. *Nature Reviews Genetics*.

[230] Fantin A, Maden CH, Ruhrberg C (2009). Neuropilin ligands in vascular and neuronal patterning. *Biochemical Society Transactions*.

[231] Mutter WP, Karumanchi SA (2008). Molecular mechanisms of preeclampsia. *Microvascular Research*.

[232] Page NM (2002). The endocrinology of pre-eclampsia. *Clinical Endocrinology*.

[233] Du H, Li P, Pan Y (2010). Vascular endothelial growth factor signaling implicated in neuroprotective effects of placental growth factor in an in vitro ischemic model. *Brain Research*.

[234] Schänzer A, Wachs F-P, Wilhelm D (2004). Direct stimulation of adult neural stem cells in vitro and neurogenesis in vivo by vascular endothelial growth factor. *Brain Pathology*.

[235] Kiuchi T, Lee H, Mikami T (2012). Regular exercise cures depression-like behavior via VEGF-Flk-1 signaling in chronically stressed mice. *Neuroscience*.

[236] Damsky CH, Fisher SJ (1998). Trophoblast pseudo-vasculogenesis: faking it with endothelial adhesion receptors. *Current Opinion in Cell Biology*.

[237] Zhou Y, Bellingard V, Feng K-T, McMaster M, Fisher SJ (2003). Human cytotrophoblasts promote endothelial survival and vascular remodeling through secretion of Ang2, PlGF, and VEGF-C. *Developmental Biology*.

[238] Hertig A, Berkane N, Lefevre G (2004). Maternal serum sFlt1 concentration is an early and reliable predictive marker of preeclampsia. *Clinical Chemistry*.

[239] Thadhani R, Kisner T, Hagmann H (2011). Pilot study of extracorporeal removal of soluble Fms-like tyrosine kinase 1 in preeclampsia. *Circulation*.

[240] Roberts JM, Cooper DW (2001). Pathogenesis and genetics of pre-eclampsia. *The Lancet*.

